# Application of K-Means Clustering for Job Applicant Analysis in Construction Firms Using R

**DOI:** 10.12688/f1000research.172383.1

**Published:** 2025-12-10

**Authors:** Daniel Jesayanto Jaya, Wahyu Muhammad Ramdhani, Endang Wati, Yogi Novario Nandes, Ilma Zahriyatun Nadhiroh, Reza Bakhrun Fidianto Pade

**Affiliations:** 1 Technology and Vocational Education and Training, Universitas Negeri Yogyakarta, Yogyakarta, Special Region of Yogyakarta, 55282, Indonesia; 2 Building Engineering Education, Universitas Negeri Jakarta, East Jakarta, Special Capital Region of Jakarta, Indonesia; 3 Educational Research and Evaluation, Universitas Negeri Yogyakarta, Yogyakarta, Special Region of Yogyakarta, 55282, Indonesia; 4 English Language Education, Universitas Negeri Yogyakarta, Yogyakarta, Special Region of Yogyakarta, 55282, Indonesia

**Keywords:** K-Means Clustering; data-driven recruitment; workforce selection; cluster visualization; construction competencies

## Abstract

This study applies the K-Means Clustering algorithm to analyse job applicant data at a construction consulting firm. Considering three main variables—AutoCAD drawing skills, planning and supervision report writing skills, and adaptability—this study aims to categorize job applicants into three groups: “Rejected,” “Under Consideration,” and “Accepted.” The clustering process was conducted using the R program with an initial centroid-based approach and visualized through 2D and 3D scatter plots to map the distribution patterns of applicants based on their attributes. The results of the study show that this clustering not only provides a deep understanding of the characteristics of applicants but also supports the company in optimizing data-driven recruitment strategies. Applicants with high scores in all three variables tend to fall into the “Accepted” category, while those with moderate scores are categorized as “Under Consideration.” The visualization of results offers sharper insights into applicant distribution, which can be used to identify training needs or improvements to the selection process. This research contributes to the development of an efficient and objective data-driven recruitment system in the construction sector.

## 1. Introduction

### 1.1 Research background

In the modern workplace, an effective workforce selection process is one of the key factors in supporting human resource development. Career transformation, as part of this development, is influenced not only by technical skills but also by an individual’s adaptability and interpersonal competencies. As explained by Pala (2021), data-driven workforce characteristic grouping can provide deep insights into how workers adapt to various work environments. The recruitment process not only involves searching for candidates from external sources but also requires effective and efficient decision-making, considering recruitment sources, techniques used, and the potential of available local labor ( Widodo, 2018). Recruitment begins with job analysis, which helps describe the tasks, responsibilities, and qualification requirements for each position, thereby facilitating the selection process and ensuring the suitability of applicants for the positions offered ( Widodo, 2018).

In the context of job searching, individuals need to understand their personal assets, study job opportunities, develop career plans, and build networks to obtain jobs that match their skills and needs ( London, 1973). This process reflects the importance of self-development to compete in an increasingly complex job market. Gangl (2003) emphasizes that job skills are the primary factor determining an individual’s chances of securing employment, particularly in sectors requiring specific technical expertise. In the construction industry, technical skills such as the ability to use AutoCAD for drafting, prepare planning reports, and adapt to collaborate with various stakeholders are highly valued attributes.

The increase in infrastructure development in Indonesia, such as the Nusantara Capital City ( *Ibu Kota Negara,* here-after IKN *)* project in East Kalimantan, is driving the need for competent construction workers. This national strategic project aims not only to relocate the country’s administrative functions from Jakarta but also to promote economic equality and development outside Java ( Irmawan et al., 2023). The IKN project requires over 260,000 construction workers by 2024, spanning various educational levels and backgrounds, to meet the demands of technical work and social adaptation on-site ( Supriyanti et al., 2023). However, the labor selection process for large projects like this often faces challenges in managing applicant data efficiently, given the large and diverse volume of data.

The cluster analysis-based approach offers a solution to help companies understand the characteristics of job applicants. Clustering is a statistical technique used to divide data into several groups based on the similarity of certain attributes, thereby facilitating interpretation and decision-making ( Jain et al., 1999). One of the most commonly used clustering algorithms is K-Means Clustering, which divides data into several clusters based on the distance to the centroid, which is the center of the data. This algorithm works through iterations to minimize the distance between the data and the cluster centroid until an optimal result is achieved. In the context of workforce selection, K-Means allows companies to group applicants into specific categories such as Rejected, Under Consideration, and Accepted, based on their specific characteristics.

This process also opens up opportunities to identify applicants with the highest potential for further development, as outlined in the literature on career transformation that emphasizes mapping individual competency strengths ( El Achmar & Bhagat, 2023). Through this mapping, companies can not only screen candidates more objectively but also guide future workforce development. The 2D and 3D scatter plots generated from this clustering provide an intuitive visual representation, making it easier to make data-driven decisions. This research is unique because it applies the K-Means algorithm directly to job applicant data obtained from a construction consulting company. The data includes three main variables: ability to draw using AutoCAD, ability to create planning and supervision reports, and social adaptability. 2D and 3D scatter plots are used to visualize the clustering results, providing deeper insights into the distribution patterns of job applicants in the construction sector.

Previous studies have demonstrated the effectiveness of the K-Means algorithm in various applications. Chen Yu (2020) explains that K-Means can help identify relevant patterns and trends in various fields, including education and industry. Wiharto and Suryani (2020) compared the K-Means algorithm with Fuzzy C-Means and found that K-Means is more efficient in terms of computation time. Additionally, Agustina and Prihandoko (2018) applied K-Means to group employees based on their level of discipline and performance, providing a basis for decision-making in human resource management.

Through this research, it is hoped that the clustering results will not only help companies screen job applicants more objectively but also serve as a basis for developing data-driven recruitment policies. This research also provides insights that can be utilized by educational institutions such as vocational schools and universities to align their curricula with the specific needs of the construction sector. Thus, this research contributes to enhancing the efficiency and accuracy of workforce selection while strengthening the connection between education and the construction industry, particularly in addressing the significant challenges of national development.

### 1.2 Literature review

Clustering is a technique used to group objects into specific clusters based on attribute similarities. Objects grouped into one cluster have a high degree of similarity compared to objects in other clusters. This process enables the separation of data regions so that objects with similar characteristics are in the same cluster region ( Jain et al., 1999). With this method, data variation within a group can be minimized, while differences between groups can be maximized, thereby facilitating data analysis and interpretation. Clustering is a statistical technique used to group objects into specific groups based on attribute similarity. Objects within a cluster have a higher degree of relatedness compared to objects in other clusters ( Manikandan et al., 2018). By grouping data or objects, variation within a group can be minimized, while differences between groups can be maximized. This process facilitates the interpretation of complex data, provides deeper insights, and aids in data-driven decision-making ( Darmi & Setiawan, 2016).

One of the most commonly used clustering methods is K-Means Clustering, which works by dividing data into several clusters based on attribute similarities. This method uses centroids as the center of data in each cluster. The position of the centroid will continue to be updated through iterations until stability is achieved, to minimize the distance between each data point and the cluster centroid ( Jain et al., 1999). Fadhli (2017) explains that K-Means is very effective in clustering high-dimensional data, making it a powerful tool for understanding and visualizing complex data. In the context of education, Firza and Sarjono (2020) show that the K-Means algorithm can be applied to analyze student data, such as major preferences and learning achievement evaluations.

In previous research, Wiharto and Suryani (2020) compared the K-Means algorithm with Fuzzy C-Means. The results showed that K-Means was more efficient in terms of computation time, although Fuzzy C-Means could produce higher accuracy in certain cases. Additionally, Agustina and Prihandoko (2018) applied K-Means to cluster employees based on discipline levels and performance, providing a basis for decision-making in human resource management. These studies demonstrate the flexibility of the K-Means algorithm across various applications, including in the fields of education and industry.

1.2.1 K-Means algorithm

K-Means is one of the partitioning clustering methods used in data analysis to divide data into several groups based on similar characteristics. This clustering process is performed iteratively, to minimize the average distance between each data point and its cluster center (centroid). K-Means is highly effective in grouping data because it can create clusters with a high degree of similarity within the group while distinguishing them from other clusters ( Widiyaningtyas et al., 2017).

The K-Means algorithm process begins by determining the desired number of clusters (k). Next, the cluster center or initial centroid is selected randomly to start the iteration. After the first iteration, the centroid value is updated based on the average position of the members in the cluster until the centroid position is stable. The stages of the K-Means algorithm process are generally as follows ( Purba et al., 2018):

a. Determine the number of clusters
  The first step in the K-Means algorithm is to determine the number of clusters to be formed based on the analysis requirements. The value of k is the main parameter that must be set at the beginning of the process.
b. Determining the initial centroid value
  The centroid, or cluster center point, for the initial iteration is selected randomly as the first step in clustering. Once the iteration begins, the centroid position will be recalculated using the following formula.
$$\bar{v_{\mathrm{ij}}}=\frac{1}{N_{i}}\sum_{i=0}^{N_{i}}x_{\mathrm{ij}}$$

Explanation:

$\bar{v_{\mathrm{ij}}}$ = centroid or average for cluster i on the variable j

$N_{i}$ = number of data points belonging to a cluster

i = cluster index

j = variable index

$x_{\mathrm{ij}}$ = data value i in the cluster for variable j

c. Calculate the distance between the centroid and the object
  This stage calculates the distance between each data point and the cluster centroid using Euclidean Distance, which is formulated as:
$$D_{e}=\sqrt{{\left(x_{i}-s_{i}\right)}^{2}+{\left(y_{i}-t_{i}\right)}^{2}}$$

Explanation:

$D_{e}$ = distance between objects and centroid

x,y = object coordinates

s,t = centroid coordinates

i = the number of objects

d. Grouping objects into clusters
  Object data will be allocated to clusters based on the minimum distance to the centroid. Each object will have a membership value in the distance matrix, with a value of 1 if the data is allocated to a specific cluster, and 0 if it is allocated to another cluster. After the objects are grouped, the algorithm will return to the second stage to recalculate the centroid position. This process is repeated until the centroid position no longer changes and the data does not move between clusters, indicating that the grouping process is complete.
e. Repeat steps 3 and 4
  The purpose of this repetition is to achieve a centroid position that no longer changes or to find that the centroid position difference is below the specified threshold.

1.2.2 Worker recruitment

Recruitment is a strategic process carried out by companies to obtain quality workers in line with organizational needs. This process involves searching for candidates from internal and external sources and making effective decisions based on job analysis. Job analysis is an important first step in describing the tasks, responsibilities, and qualifications required for each position, thereby helping to match applicants with company needs ( Widodo, 2018).

According to Smith and Todaro (2015), labor absorption is greatly influenced by the availability of jobs and the quality of human resources. Technical competencies such as the ability to draw using AutoCAD, prepare planning reports, and adaptability are highly valued attributes in the construction sector ( Gangl, 2003). Additionally, Green et al. (2011) state that the job search process aims to match job seekers with suitable job opportunities, which can be facilitated through technology or data-driven methods.

In facing the challenges of managing large and diverse applicant data, algorithms such as K-Means Clustering can be a solution to simplify the analysis of job applicant data. This method divides data into clusters based on attribute similarities, enabling companies to categorize applicants into groups such as Rejected, Under Consideration, and Accepted ( Jain et al., 1999). With this data-driven approach, recruitment can be conducted more efficiently and objectively, supporting companies in selecting the best candidates for their needs.

## 2. Methods

The research methodology consists of several stages designed to collect and analyze data on job applicants for the Construction Consulting Company.

**Figure 1.**
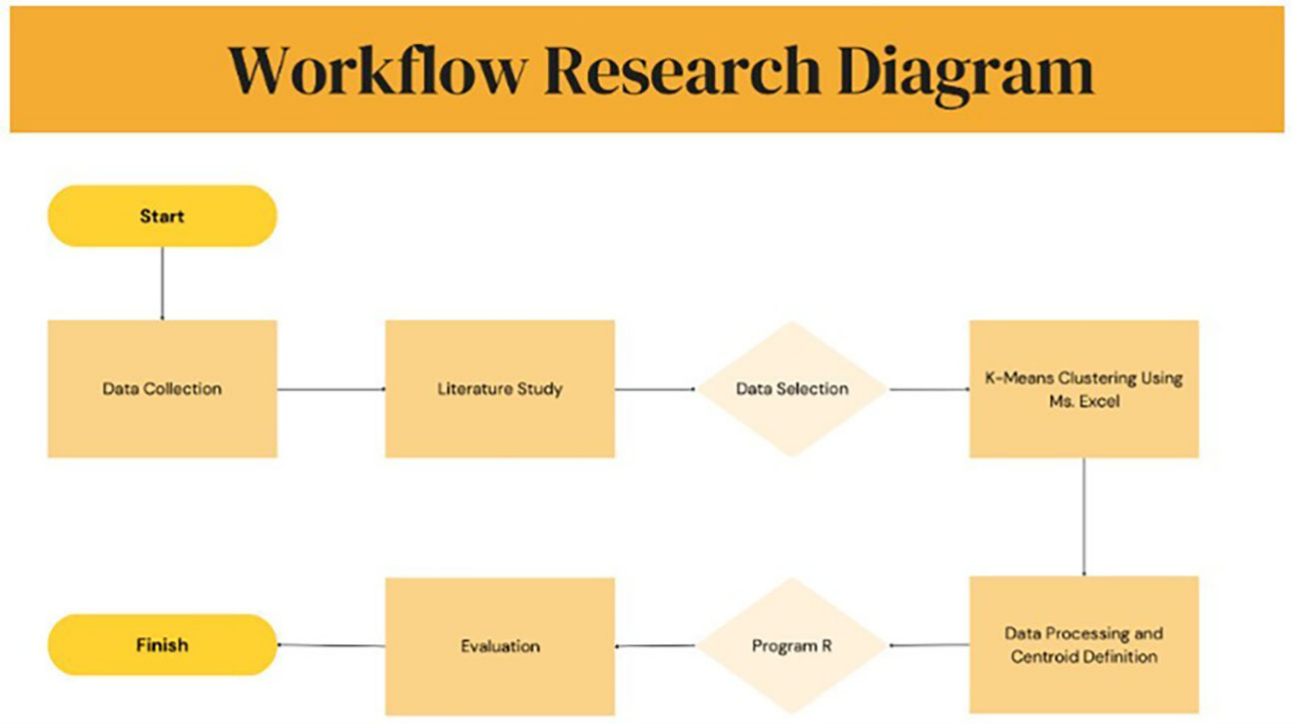
Workflow research diagram. This figure illustrates the overall research workflow, including data collection, literature review, data selection, centroid initialization, K-Means clustering (Excel), and subsequent evaluation and visualization using R software.

The overall workflow for data collection, preprocessing, clustering, and evaluation is summarized in Figure 1. This research methodology is designed to analyse job applicant data at a construction consulting firm using the K-Means Clustering algorithm. The research process begins with the collection of information and a literature review on K-Means Clustering, the use of the R program, and an analysis of relevant skills in the construction sector. The literature used includes reliable sources such as Google Scholar, research journals, and reference books.

Data collection was conducted directly from construction consulting companies, covering applicants’ ability to draw using AutoCAD, their ability to compile planning reports, and their social adaptability. Irrelevant or incomplete data was filtered through a data cleaning process to ensure the accuracy of the analysis. According to Kassambara (2017), the data cleaning step is very important in cluster analysis to produce valid and reliable results.

In this study, the K-Means Clustering-based clustering method was applied to analyze job applicant data. This algorithm is highly relevant due to its ability to group applicants based on characteristics such as drawing skills, planning reports, and adaptability. As explained by Lynn et al. (2023), this type of clustering approach is often used in the context of career development to map individuals into groups based on similar attributes.

The K-Means algorithm was applied using the R program. The number of clusters was determined using the elbow method, which is a common technique for determining the optimal number of clusters in K-Means analysis. The clustering process involved calculating the Euclidean distance between the data and the cluster centroid, with iterations continuing until the cluster center stabilized. The clustering results are then evaluated using the Davies-Bouldin index to assess the validity and quality of the clusters ( Gie & Jollyta, 2020). This evaluation aims to ensure that the resulting grouping is relevant and appropriate for the analysis needs.

The clustering results are visualized using 2D and 3D scatter plots to provide a clear picture of the distribution of applicants in each cluster. Further analysis is conducted to understand the characteristics of each cluster, including the dominant abilities and work preferences of applicants. With this approach, construction consulting companies can be more effective in developing data-driven recruitment strategies.

The final stage of this research is the preparation of a report containing findings, conclusions, and recommendations. This report is expected to make a significant contribution to companies in improving the efficiency of workforce selection, as well as supporting the construction sector in facing national development challenges.

## 3. Results and discussion

Data were obtained from CV Ardantama Putra Perkasa as part of their internal recruitment records. The company had posted a job vacancy on JobStreet Indonesia as the external recruitment channel; however, all applicant data used in this study originated from the company’s internal testing and selection process, not from the JobStreet platform.

In total, 161 applicants applied to the vacancy, and 30 candidates who met the minimum requirements were invited for in-person testing. The assessment consisted of three evaluation components: AutoCAD Drawing Skills (X), Ability to Prepare Planning and Supervision Reports (Y), and Adaptability (Z). Descriptive information for the 30 tested applicants is provided in Table 1.

**Table 1.** Applicant demographic data.

Respondent code	Gender	AutoCAD drawing skills (X)	Ability to prepare planning and monitoring reports (Y)	Adaptability (Z)
Resp1	Female	92	75	68
Resp2	Male	68	65	66
Resp3	Male	73	86	87
Resp4	Male	69	74	73
Resp5	Male	78	72	91
Resp6	Female	84	90	92
Resp7	Male	69	76	87
Resp8	Female	95	73	76
Resp9	Female	90	80	85
Resp10	Male	68	82	68
Resp11	Male	63	75	71
Resp12	Male	75	93	77
Resp13	Female	62	72	68
Resp14	Male	90	61	72
Resp15	Female	84	63	90
Resp16	Female	94	70	89
Resp17	Female	73	87	80
Resp18	Female	71	73	95
Resp19	Female	93	62	70
Resp20	Male	90	68	89
Resp21	Female	87	94	87
Resp22	Male	60	90	64
Resp23	Female	65	64	93
Resp24	Male	69	84	75
Resp25	Male	66	63	72
Resp26	Male	95	85	93
Resp27	Male	75	80	83
Resp28	Male	92	85	93
Resp29	Male	71	71	85
Resp30	Male	92	61	88

With this data, the objects were grouped into three clusters with the attributes Rejected, Under Consideration, and Accepted. Next, determine the Centroid Value. The centroid value is chosen randomly, as follows: Rejected = (60,75,85), Considered = (62,77,88), Accepted = (70,84,92). The next step is to calculate the distance based on the determined Centroid point. Initial centroid selection and the corresponding Euclidean distances for each applicant are reported in Table 2.

**Table 2.** Initial centroid distances.

Respondent data				Rejected			Under consideration			Accepted		
				C1(x _1_,y _1_,z _1_)			C2(x _2_,y _2_,z _2_)			C3(x _3_,y _3_,z _3_)		
Name	MA	LPP	KA	60	75	85	62	77	88	70	84	92
Resp1	92	75	68	36,24			36,11			33,78		
Resp2	68	65	66	22,91			25,77			32,26		
Resp3	73	86	87	17,15			14,25			6,16		
Resp4	69	74	73	15,03			16,82			21,49		
Resp5	78	72	91	19,21			17,03			14,46		
Resp6	84	90	92	29,15			25,87			15,23		
Resp7	69	76	87	9,27			7,14			9,49		
Resp8	95	73	76	36,19			35,34			31,65		
Resp9	90	80	85	30,41			28,32			21,56		
Resp10	68	82	68	20,05			21,47			24,17		
Resp11	63	75	71	14,32			17,15			23,90		
Resp12	75	93	77	24,76			23,37			18,19		
Resp13	62	72	68	17,38			20,62			28,00		
Resp14	90	61	72	35,57			36,00			36,46		
Resp15	84	63	90	27,29			26,15			25,32		
Resp16	94	70	89	34,60			32,77			27,95		
Resp17	73	87	80	18,38			16,88			12,73		
Resp18	71	73	95	15,00			12,08			11,45		
Resp19	93	62	70	38,51			38,86			38,69		
Resp20	90	68	89	31,06			29,43			25,79		
Resp21	87	94	87	33,08			30,25			20,35		
Resp22	60	90	64	25,81			27,37			30,33		
Resp23	65	64	93	14,49			14,25			20,64		
Resp24	69	84	75	16,19			16,34			17,03		
Resp25	66	63	72	18,68			21,63			29,27		
Resp26	95	85	93	37,27			34,32			25,04		
Resp27	75	80	83	15,94			14,25			11,05		
Resp28	92	85	93	34,47			31,45			22,05		
Resp29	71	71	85	11,70			11,22			14,80		
Resp30	92	61	88	35,06			34,00			32,08		

After calculating the distance matrix by entering the formula:

$$C_{1}=\sqrt{{\left(60+92\right)}^{2}+{\left(75+75\right)}^{2}+{\left(85+68\right)}^{2}}=36,24$$

$$C_{2}=\sqrt{{\left(62+92\right)}^{2}+{\left(77+75\right)}^{2}+{\left(88+68\right)}^{2}}=36,11$$

$$C_{3}=\sqrt{{\left(70+92\right)}^{2}+{\left(84+75\right)}^{2}+{\left(92+68\right)}^{2}}=33,78$$

This formula is repeated or iterated until the 30th data point is filled, then the cluster members are determined according to the minimum distance from the centroid. Referring to the distance matrix, this can be seen in the values obtained in Table 3 above, which are colored red for Cluster 1, yellow for Cluster 2, and green for Cluster 3. Next, each cluster is combined or grouped into one cluster, and the second centroid point and its distance are calculated. Updated cluster memberships and second-stage centroid distances are summarized in Table 3.

**Table 3.** Second-stage centroid values and distances.

Respondent data				Rejected			Under consideration			Accepted		
				C1(x _1_,y _1_,z _1_)			C2(x _2_,y _2_,z _2_)			C3(x _3_,y _3_,z _3_)		
Name	MA	LPP	KA	70,80	72,80	69,90	68,33	70,33	88,33	84,71	78,53	86,06
Resp2	68	65	66	9,16			22,96			29,40		
Resp4	69	74	73	3,78			15,78			20,92		
Resp10	68	82	68	9,80			23,44			24,84		
Resp11	63	75	71	8,18			18,73			26,65		
Resp13	62	72	68	9,04			21,36			29,74		
Resp14	90	61	72	22,63			28,69			23,09		
Resp19	93	62	70	24,69			31,84			24,49		
Resp22	60	90	64	21,15			32,38			35,05		
Resp24	69	84	75	12,44			19,10			19,97		
Resp25	66	63	72	11,11			18,06			28,08		
Resp7	69	76	87	17,49			5,86			15,94		
Resp23	65	64	93	25,39			8,54			25,45		
Resp29	71	71	85	15,21			4,32			15,67		
Resp1	92	75	68	21,40			31,55			19,79		
Resp3	73	86	87	21,71			16,40			13,92		
Resp5	78	72	91	22,31			10,17			10,58		
Resp6	84	90	92	30,96			25,41			12,94		
Resp8	95	73	76	24,96			29,50			15,42		
Resp9	90	80	85	25,47			23,96			5,60		
Resp12	75	93	77	21,82			26,20			19,64		
Resp15	84	63	90	25,97			17,38			16,04		
Resp16	94	70	89	30,18			25,68			12,95		
Resp17	73	87	80	17,56			19,21			15,67		
Resp18	71	73	95	25,10			7,66			17,27		
Resp20	90	68	89	27,50			21,80			12,15		
Resp21	87	94	87	31,69			30,17			15,67		
Resp26	95	85	93	35,61			30,79			14,00		
Resp27	75	80	83	15,53			12,90			10,28		
Resp28	92	85	93	33,64			28,23			11,97		
Resp30	92	61	88	30,27			25,44			19,09		

Based on the table above, the results of the calculation of the centroid point and distance point for the second stage have been obtained. It turns out that there is still a shift in cluster data, so it is necessary to calculate the centroid point and distance point for the third stage. The third-stage centroid updates and distances are presented in Table 4.

**Table 4.** Third-stage centroid values.

Respondent data				Rejected			Under consideration			Accepted		
				C1(x _1_,y _1_,z _1_)			C2(x _2_,y _2_,z _2_)			C3(x _3_,y _3_,z _3_)		
Name	MA	LPP	KA	68,33	74,00	69,89	70,80	71,20	90,20	86,50	78,25	84,19
Resp2	68	65	66	9,81			25,14			29,13		
Resp4	69	74	73	3,18			17,52			21,20		
Resp10	68	82	68	8,23			24,85			24,87		
Resp11	63	75	71	5,54			21,07			27,14		
Resp13	62	72	68	6,90			23,89			30,02		
Resp14	90	61	72	25,36			28,35			21,41		
Resp22	60	90	64	18,98			34,01			35,32		
Resp24	69	84	75	11,25			19,95			20,58		
Resp25	66	63	72	11,44			20,53			28,31		
Resp7	69	76	87	17,24			6,04			17,87		
Resp23	65	64	93	25,40			9,66			27,26		
Resp29	71	71	85	15,64			5,21			17,13		
Resp5	78	72	91	23,31			7,29			12,56		
Resp18	71	73	95	25,27			5,13			19,61		
Resp19	93	62	70	27,43			31,39			22,53		
Resp1	92	75	68	23,76			30,93			17,40		
Resp3	73	86	87	21,41			15,30			15,82		
Resp6	84	90	92	31,47			23,04			14,33		
Resp8	95	73	76	27,38			28,12			12,92		
Resp9	90	80	85	27,09			21,75			4,00		
Resp12	75	93	77	21,35			25,83			20,04		
Resp15	84	63	90	27,77			15,54			16,51		
Resp16	94	70	89	32,25			23,26			12,14		
Resp17	73	87	80	17,12			18,93			16,62		
Resp20	90	68	89	29,51			19,50			11,85		
Resp21	87	94	87	32,27			28,15			16,01		
Resp26	95	85	93	36,96			28,00			13,98		
Resp27	75	80	83	15,89			12,12			11,69		
Resp28	92	85	93	34,86			25,45			12,39		
Resp30	92	61	88	32,51			23,63			18,50		

Based on the table above, the results of the calculation of the centroid point and distance point for the third stage have been obtained, but it turns out that there is still a shift in cluster data, so it is necessary to calculate the centroid point and distance point for the fourth stage. The fourth-stage centroid updates and distances are presented in Table 5.

**Table 5.** Fourth-stage centroid values.

Respondent data				Rejected			Under consideration			Accepted		
				C1(x _1_,y _1_,z _1_)			C2(x _2_,y _2_,z _2_)			C3(x _3_,y _3_,z _3_)		
Name	MA	LPP	KA	65,63	75,63	69,63	73,00	72,14	89,71	87,80	77,60	82,80
Resp2	68	65	66	11,47			25,27			28,86		
Resp4	69	74	73	5,04			17,29			21,50		
Resp10	68	82	68	6,99			24,37			25,11		
Resp11	63	75	71	3,03			21,41			27,59		
Resp13	62	72	68	5,38			24,34			30,27		
Resp22	60	90	64	16,43			33,90			35,78		
Resp24	69	84	75	10,51			19,32			21,34		
Resp25	66	63	72	12,85			21,13			28,37		
Resp7	69	76	87	17,70			6,18			19,33		
Resp23	65	64	93	26,11			11,88			28,44		
Resp29	71	71	85	16,93			5,25			18,18		
Resp5	78	72	91	24,96			5,16			13,95		
Resp18	71	73	95	26,07			5,72			21,27		
Resp3	73	86	87	21,54			14,12			17,53		
Resp15	84	63	90	30,20			14,31			16,72		
Resp14	90	61	72	28,52			26,96			19,93		
Resp19	93	62	70	30,58			29,86			20,84		
Resp1	92	75	68	26,43			28,99			15,60		
Resp6	84	90	92	32,33			21,10			15,90		
Resp8	95	73	76	30,17			25,94			10,92		
Resp9	90	80	85	29,15			19,31			3,93		
Resp12	75	93	77	21,08			24,51			20,85		
Resp16	94	70	89	34,82			21,12			11,60		
Resp17	73	87	80	17,07			17,75			17,75		
Resp20	90	68	89	32,06			17,51			11,64		
Resp21	87	94	87	33,11			26,10			16,95		
Resp26	95	85	93	38,69			25,69			14,51		
Resp27	75	80	83	16,91			10,53			13,02		
Resp28	92	85	93	36,47			23,18			13,28		
Resp30	92	61	88	35,32			22,09			17,90		

Based on the table above, the results of the calculation of the centroid point and distance point for the fourth stage were obtained, but it was found that there was still a shift in the cluster data, so it was necessary to calculate the centroid point and distance point for the fifth stage. The fifth-stage centroid updates and distances are presented in Table 6.

**Table 6.** Fifth-stage centroid values.

Respondent data				Rejected			Under consideration			Accepted		
				C1(x _1_,y _1_,z _1_)			C2(x _2_,y _2_,z _2_)			C3(x _3_,y _3_,z _3_)		
Name	MA	LPP	KA	66,44	76,89	70,78	73,25	73,13	88,88	89,92	76,69	83,00
Resp2	68	65	66	12,91			24,84			30,11		
Resp4	69	74	73	4,45			16,46			23,35		
Resp10	68	82	68	6,02			23,28			27,09		
Resp11	63	75	71	3,93			20,69			29,52		
Resp13	62	72	68	7,17			23,74			32,04		
Resp22	60	90	64	16,10			32,85			37,86		
Resp24	69	84	75	8,66			18,13			23,56		
Resp25	66	63	72	13,95			20,97			29,68		
Resp17	73	87	80	15,17			16,47			20,04		
Resp7	69	76	87	16,45			5,46			21,31		
Resp23	65	64	93	25,73			12,97			29,70		
Resp29	71	71	85	16,05			4,96			19,86		
Resp5	78	72	91	23,80			5,32			15,11		
Resp18	71	73	95	24,95			6,53			22,71		
Resp3	73	86	87	19,73			13,01			19,72		
Resp15	84	63	90	29,51			14,81			16,48		
Resp27	75	80	83	15,24			9,21			15,29		
Resp14	90	61	72	28,44			26,69			19,16		
Resp19	93	62	70	30,45			29,50			19,86		
Resp1	92	75	68	25,78			28,12			15,24		
Resp6	84	90	92	30,50			20,25			17,12		
Resp8	95	73	76	29,29			25,28			9,40		
Resp9	90	80	85	27,69			18,52			3,87		
Resp12	75	93	77	19,27			23,22			22,91		
Resp16	94	70	89	33,75			20,98			9,87		
Resp20	90	68	89	31,08			17,52			10,56		
Resp21	87	94	87	31,28			25,07			18,00		
Resp26	95	85	93	37,08			25,12			13,96		
Resp28	92	85	93	34,82			22,57			13,17		
Resp30	92	61	88	34,67			22,35			16,60		

Based on the table above, the results of the centroid point and distance point calculations for the fifth stage were obtained, but it was found that there was still a shift in the cluster data, so it was necessary to calculate the centroid point and distance point for the sixth stage. The sixth-stage centroid updates and distances are presented in Table 7.

**Table 7.** Sixth-stage centroid values.

Respondent data				Rejected			Under consideration			Accepted		
				C1(x _1_,y _1_,z _1_)			C2(x _2_,y _2_,z _2_)			C3(x _3_,y _3_,z _3_)		
Name	MA	LPP	KA	67,30	78,50	71,40	73,25	73,13	88,88	91,17	75,33	83,50
Resp2	68	65	66	14,56			24,84			30,82		
Resp4	69	74	73	5,07			16,46			24,56		
Resp10	68	82	68	4,93			23,28			28,66		
Resp11	63	75	71	5,56			20,69			30,82		
Resp13	62	72	68	9,05			23,74			33,20		
Resp22	60	90	64	15,50			32,85			39,58		
Resp24	69	84	75	6,79			18,13			25,27		
Resp25	66	63	72	15,57			20,97			30,29		
Resp17	73	87	80	13,37			16,47			21,87		
Resp12	75	93	77	17,35			23,22			24,81		
Resp7	69	76	87	15,89			5,46			22,45		
Resp23	65	64	93	26,12			12,97			30,06		
Resp29	71	71	85	15,97			4,96			20,68		
Resp5	78	72	91	23,26			5,32			15,52		
Resp18	71	73	95	24,51			6,53			23,33		
Resp3	73	86	87	18,22			13,01			21,36		
Resp15	84	63	90	29,41			14,81			15,68		
Resp27	75	80	83	14,00			9,21			16,83		
Resp14	90	61	72	28,67			26,69			18,41		
Resp19	93	62	70	30,57			29,50			19,06		
Resp1	92	75	68	25,18			28,12			15,53		
Resp6	84	90	92	28,91			20,25			18,40		
Resp8	95	73	76	28,61			25,28			8,74		
Resp9	90	80	85	26,50			18,52			5,04		
Resp16	94	70	89	33,09			20,98			8,17		
Resp20	90	68	89	30,58			17,52			9,24		
Resp21	87	94	87	29,52			25,07			19,44		
Resp26	95	85	93	35,72			25,12			14,09		
Resp28	92	85	93	33,45			22,57			13,58		
Resp30	92	61	88	34,52			22,35			15,05		

Based on the table above, red represents Cluster 1, which represents the group of rejected individuals, yellow represents Cluster 2, which represents the group of individuals under consideration, and green represents Cluster 3, which represents the group of accepted individuals. Final cluster assignments for all applicants are listed in Table 8.

**Table 8.** Final clustering results.

Respondent data				Rejected			Under consideration			Accepted			Clustering
				C1(x _1_,y _1_,z _1_)			C2(x _2_,y _2_,z _2_)			C3(x _3_,y _3_,z _3_)			
Name	MA	LPP	KA	67,30	78,50	71,40	73,25	73,13	88,88	91,17	75,33	83,50	
Resp2	68	65	66	14,56			24,84			30,82			Cluster 1 (Rejected)
Resp4	69	74	73	5,07			16,46			24,56			
Resp10	68	82	68	4,93			23,28			28,66			
Resp11	63	75	71	5,56			20,69			30,82			
Resp13	62	72	68	9,05			23,74			33,20			
Resp22	60	90	64	15,50			32,85			39,58			
Resp24	69	84	75	6,79			18,13			25,27			
Resp25	66	63	72	15,57			20,97			30,29			
Resp17	73	87	80	13,37			16,47			21,87			
Resp12	75	93	77	17,35			23,22			24,81			
Resp7	69	76	87	15,89			5,46			22,45			Cluster 2 (Under Consideration)
Resp23	65	64	93	26,12			12,97			30,06			
Resp29	71	71	85	15,97			4,96			20,68			
Resp5	78	72	91	23,26			5,32			15,52			
Resp18	71	73	95	24,51			6,53			23,33			
Resp3	73	86	87	18,22			13,01			21,36			
Resp15	84	63	90	29,41			14,81			15,68			
Resp27	75	80	83	14,00			9,21			16,83			
Resp14	90	61	72	28,67			26,69			18,41			Cluster 3 (Accepted)
Resp19	93	62	70	30,57			29,50			19,06			
Resp1	92	75	68	25,18			28,12			15,53			
Resp6	84	90	92	28,91			20,25			18,40			
Resp8	95	73	76	28,61			25,28			8,74			
Resp9	90	80	85	26,50			18,52			5,04			
Resp16	94	70	89	33,09			20,98			8,17			
Resp20	90	68	89	30,58			17,52			9,24			
Resp21	87	94	87	29,52			25,07			19,44			
Resp26	95	85	93	35,72			25,12			14,09			
Resp28	92	85	93	33,45			22,57			13,58			
Resp30	92	61	88	34,52			22,35			15,05			

The results of this study indicate that the application of K-Means Clustering to the test results of job applicants at a construction consulting company can be identified into several groups with different characteristics. After conducting the analysis, it was found that there were three (3) main clusters formed based on the test scores of the applicants. The first cluster consisted of applicants who exceeded the requirements and were declared Accepted, while the second cluster included applicants with test scores that met the criteria and were declared Under Consideration, and the third cluster was dominated by applicants who did not meet the criteria.

After obtaining Cluster data using K-Means in Excel, the next step is to code it in the R programming language to see a picture of multivariate statistics. The analysis process begins with processing the data of participants in the selection test for a construction consulting company. The dataset consists of three main variables, namely AutoCAD Drawing, Supervision Planning Report, and Adaptability, each of which is assessed on a scale of 0 to 100. These three variables describe the technical competencies and soft skills relevant to job selection. The next stage is the initiation of the initial centroid. The initial centroids were determined based on prior calculations taken from the analysis in Microsoft Excel. Three initial centroids were set to begin the clustering process. These initial centroid values represent the initial average values for the three planned cluster groups, namely clusters with Rejected, Under Consideration, and Accepted statuses. These initial centroids will be used as a reference to determine the membership of each data point in the cluster.

To group data into clusters, the k-means_manual function is used. This function works iteratively to calculate the Euclidean distance between each data point and the predetermined initial centroid. Based on the distance calculation results, each data point will be assigned to the cluster with the minimum distance from the centroid. Once cluster membership is determined, a new centroid is calculated as the average value of the data points included in that cluster. This process is repeated until the centroid stabilizes, i.e., when the difference between the new centroid and the previous centroid becomes very small (converges), or until the iteration reaches the specified maximum limit. This process does not yet produce a final output in the form of cluster labels or visualizations, but it has completed an important step in the K-Means algorithm, namely determining the optimal centroid. Thus, the distribution of data in each cluster can better reflect the patterns or structures that exist in the dataset.

At this stage, the clustering process of the data of participants in the entrance test for construction consulting companies is continued by adding the clustering results to the dataset. The clustering results, in the form of cluster numbers, are stored in a new column called Cluster. In addition, to facilitate interpretation of the results, categorization labels are added based on these cluster numbers. These labels are defined using the factor() function with three categories, namely “Rejected,” “Under Consideration,” and “Accepted,” according to the predetermined cluster order. Next, the serial number or respondent identity is added to the dataset in a new column named No, which is generated using the 1:nrow (participant_data) function. This is done to ensure that each row of data has a unique identity that facilitates the tracking of clustering results. The data is then rearranged to be more structured by only displaying relevant columns, namely the respondent number (No), variable values (AutoCAD Drawing, Planning and Supervision Report, Adaptability), cluster number (Cluster), and category label (Category). This rearrangement process is done using the select() function from the dplyr library. As a final result of this stage, the updated dataset with additional columns and a neat layout is printed to the console using the print() function. This dataset provides a clear overview of each respondent along with variable values and clustering results in a simple table format. With this format, the data can be used for further analysis or visualized as needed. The tidy R output showing the variables, cluster IDs, and category labels is shown in Table 9.

**Table 9.** R-generated data table.

No	AutoCAD_Drafting	Planning_Supervision_ Reports	Adaptability	Cluster	Category
1	68	65	66	1	Rejected
2	69	74	73	1	Rejected
3	68	82	68	1	Rejected
4	63	75	71	1	Rejected
5	62	72	68	1	Rejected
6	60	90	64	1	Rejected
7	69	84	75	2	Under Consideration
8	66	63	72	1	Rejected
9	73	87	80	2	Under Consideration
10	75	93	77	2	Under Consideration
11	69	76	87	3	Accepted
12	65	64	93	1	Rejected
13	71	71	85	2	Under Consideration
14	78	72	91	3	Accepted
15	71	73	95	2	Under Consideration
16	73	86	87	2	Under Consideration
17	84	63	90	3	Accepted
18	75	80	83	2	Under Consideration
19	90	61	72	2	Under Consideration
20	93	62	70	2	Under Consideration
21	92	75	68	2	Under Consideration
22	84	90	92	3	Accepted
23	95	73	76	3	Accepted
24	90	80	85	3	Accepted
25	94	70	89	3	Accepted
26	90	68	94	3	Accepted
27	87	84	87	3	Accepted
28	95	85	93	3	Accepted
29	92	85	93	3	Accepted
30	92	61	88	2	Under Consideration

**Figure 2.**
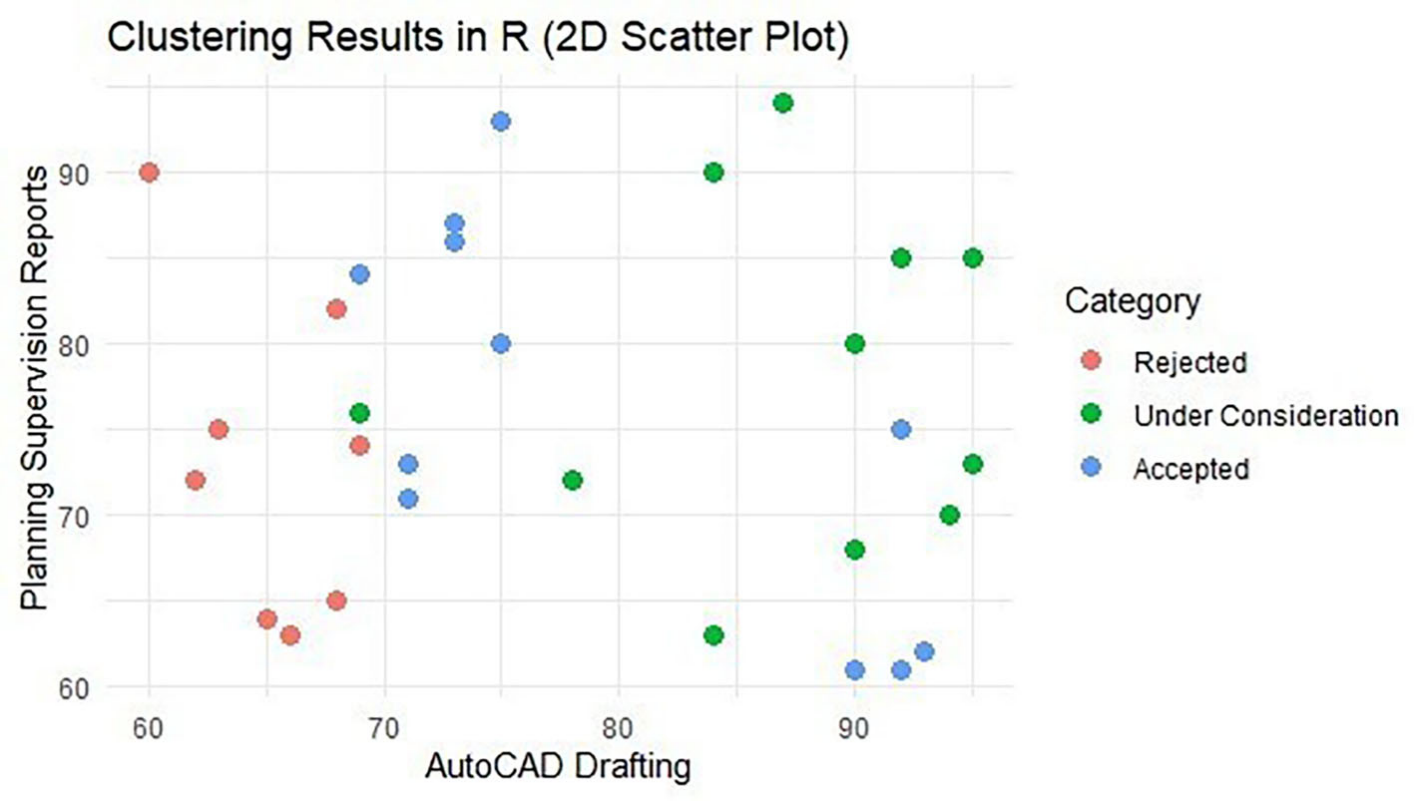
K-means clustering visualization in a 2D scatter plot. This plot displays job applicants based on AutoCAD Drawing scores (X-axis) and Planning/Supervision Report scores (Y-axis). Data points are assigned to three clusters—Rejected, Under Consideration, and Accepted—highlighting the separation of applicant competency profiles.

The image above shows the results of running the R program after initializing the data and adding several instructions to generate clusters according to the K-Means algorithm. A two-dimensional visualization of the clustering based on AutoCAD Drawing and Planning/Supervision Reports is shown in Figure 2. Then, visualize the K-Means clusters with a 2D scatter plot. This stage visualizes the clustering results in a 2D scatter plot using ggplot2. The X-axis represents AutoCAD Drawing and the Y-axis represents Supervision Planning Report, with the color of the points indicating the clustering category: Rejected, Under Consideration, or Accepted. The plot title and axis labels were added for clarity, and a minimalist theme was applied for a clean look. This visualization helps to understand the distribution of data between clusters visually. Below is an image of the visualization.

This image is a scatter plot of the clustering results using the K-Means method. The horizontal axis (X) shows the AutoCAD Drawing score, while the vertical axis (Y) represents the Supervision Planning Report score. The points on the graph are grouped into three cluster categories:

a. Red (Rejected): Indicates participants whose cluster scores are in the lowest criteria group.
b. Yellow (Under Consideration): Indicates participants with intermediate scores who are under further consideration.
c. Green (Accepted): Indicates participants with the highest scores who are in the highest cluster.

This distribution provides a visual representation of the distribution and relationship between the two main variables based on the clustering results categories. After 2D visualization, 3D visualization is performed. This visualization utilizes three main variables, namely AutoCAD Drawing as the x-axis, Supervision Planning Report as the y-axis, and Adaptability as the z-axis. The color of each data point is determined based on the cluster category that has been generated, with red for the “Rejected” category, orange for “Under Consideration,” and green for “Accepted.”

**Figure 3.**
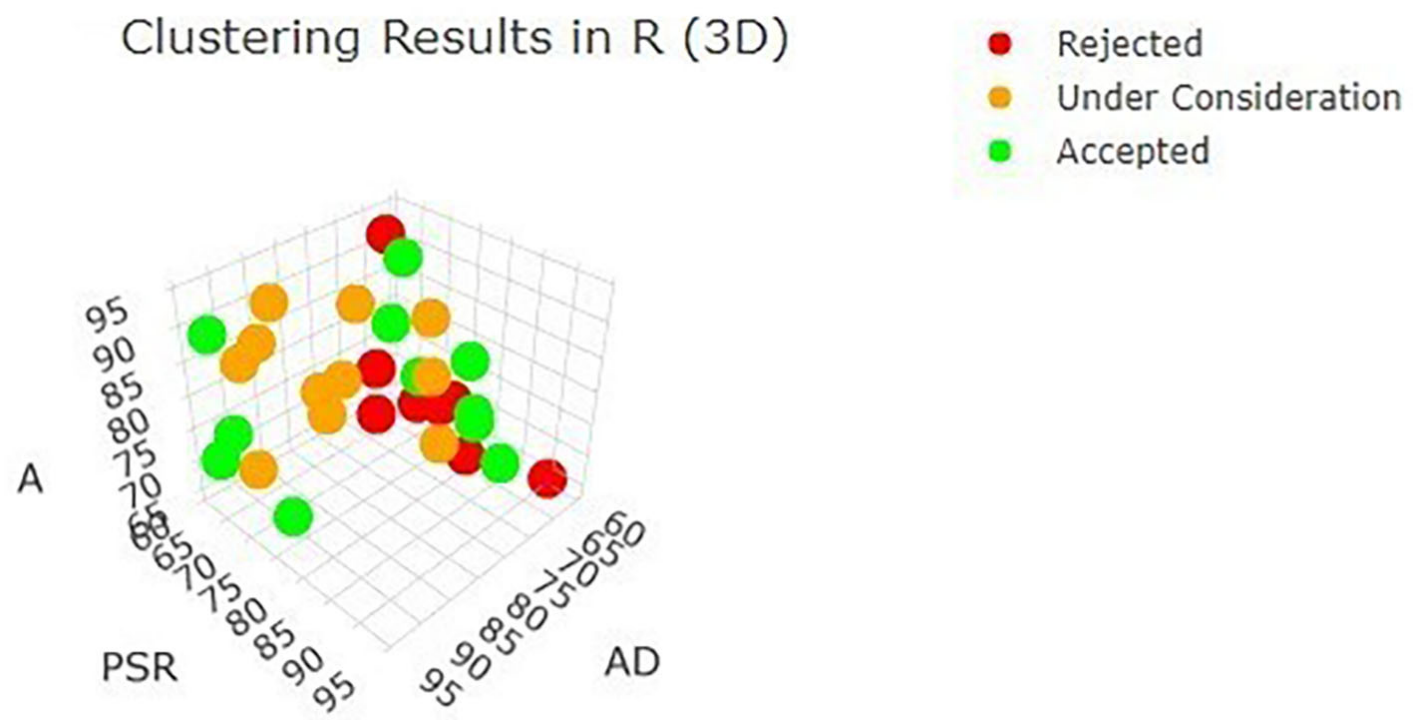
K-means clustering visualization in a 3D scatter plot. This three-dimensional plot represents AutoCAD Drawing (AD), Planning/Supervision Reports (PSR), and Adaptability (A) simultaneously. The spatial separation of the three clusters demonstrates how multivariate skill combinations distinguish applicants across the Rejected, Under Consideration, and Accepted groups.

A three-dimensional visualization using AutoCAD Drawing (X), Planning/Supervision Reports (Y), and Adaptability (Z) is displayed in Figure 3. This 3D scatter plot depicts the clustering results of selection participants based on three main variables: AutoCAD Drawing (MA), Supervision Planning Report (LPP), and Adaptability (KA). Each variable is represented on the X, Y, and Z axes, reflecting participant scores in the range of 60 to 100. This visualization maps participants into a multidimensional space to reveal distribution patterns and relationships between variables. The points in the plot are colored according to the clusterization result category: red for participants in the “Rejected” category, orange for “Under Consideration,” and green for “Accepted.” These colors facilitate interpretation by providing a quick overview of how participants are grouped based on similar characteristics.

The distribution in the graph shows that participants with high scores on all three variables tend to be in the “Accepted” category, while lower scores are more often associated with the ‘Rejected’ category. The “Under Consideration” category is in the middle, reflecting participants with moderate scores who have the possibility of moving to either category depending on additional criteria. The separation between clusters is relatively clear, indicating that clustering has successfully grouped participants with similar characteristics. Some points that appear far from the center of their cluster can be identified as outliers, providing additional information about participants with unique characteristics. This visualization provides deep insights into data structures, patterns of relationships between variables, and clustering results relevant to decision making. Next, the participants’ clusters are sorted in order. The dataset sorted by cluster and category is provided in Table 10.

**Table 10.** Sorted dataset by cluster categories.

No	AutoCAD_Drafting	Planning_Supervision_ Report	Adaptability	Cluster	Category
**1**	68	65	66	1	Rejected
**2**	69	74	73	1	Rejected
**3**	68	82	68	1	Rejected
**4**	63	75	71	1	Rejected
**5**	62	72	68	1	Rejected
**6**	60	90	64	1	Rejected
**7**	66	63	72	1	Rejected
**8**	65	64	93	1	Rejected
**9**	69	84	75	2	Under Consideration
**10**	73	87	80	2	Under Consideration
**11**	75	93	77	2	Under Consideration
**12**	71	71	85	2	Under Consideration
**13**	71	73	95	2	Under Consideration
**14**	73	86	87	2	Under Consideration
**15**	75	80	83	2	Under Consideration
**16**	90	61	72	2	Under Consideration
**17**	93	62	70	2	Under Consideration
**18**	92	75	68	2	Under Consideration
**19**	92	61	88	2	Under Consideration
**20**	69	76	87	3	Accepted
**21**	78	72	91	3	Accepted
**22**	84	63	90	3	Accepted
**23**	84	90	92	3	Accepted
**24**	95	73	76	3	Accepted
**25**	90	80	85	3	Accepted
**26**	94	70	89	3	Accepted
**27**	90	68	89	3	Accepted
**28**	87	94	87	3	Accepted
**29**	95	85	93	3	Accepted
**30**	92	85	93	3	Accepted

**Figure 4.**
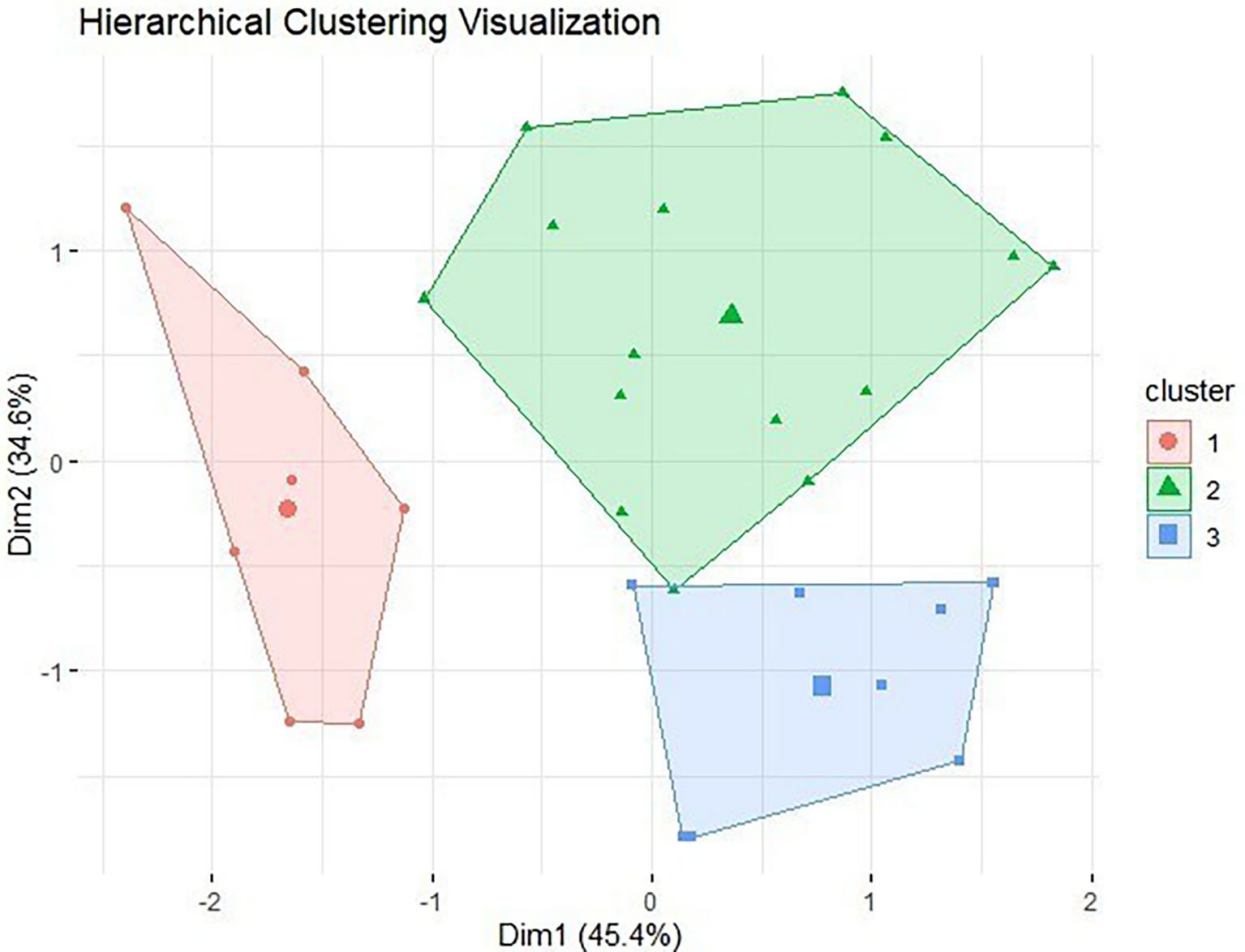
Hierarchical clustering visualization using PCA-projected dimensions. This figure maps hierarchical clustering results onto principal component space, with convex hulls outlining the boundaries of the three clusters. The grouping pattern confirms consistency between hierarchical clustering and the K-Means classification of job applicants.

The hierarchical clustering (PCA biplot with convex hulls) summarizing the separation of the three groups is depicted in Figure 4. After the data cluster sequence table appears, the hierarchical clustering results are displayed visually. Hierarchical clustering visualization is important because it helps to understand the hierarchical structure of the data, allowing the identification of relationships between groups based on distance or similarity. The resulting dendrogram facilitates analysis, such as determining the optimal number of clusters by cutting the tree at a certain level. In addition, this approach is useful for describing data in an intuitive way, especially when the relationships between data are not linear, thus providing deeper insights into the patterns or distribution of data in the dataset.

This hierarchical clustering visualization provides an overview of the grouping of construction consultant job applicants based on three main dimensions: AutoCAD Drawing (MA), Planning and Supervision Reports (LPP), and Adaptability (KA). These results illustrate the differences in characteristics among applicants in three clusters, which are relevant for determining job acceptance.

a. Cluster 1 (Red):
  i. This cluster has characteristics that are concentrated on the left side of the graph, with scores that tend to be lower than the other clusters.
  ii. Participants in this cluster are likely to have suboptimal performance on the main criteria tested.
  iii. It can be interpreted that these participants fall into the “Rejected” category, as they do not meet the minimum standards for acceptance.
b. Cluster 2 (Yellow):
  i. Located in the middle of the graph, this cluster shows participants with average performance on the main criteria.
  ii. Participants in this group have potential, but may require further evaluation or additional training to meet admission standards.
  iii. These participants fall into the “Under Consideration” category, meaning they still have a chance of being accepted.
c. Cluster 3 (Green):
  i. Located at the bottom right of the graph, this cluster consists of participants with higher scores across all dimensions.
  ii. These participants demonstrate the best and most consistent performance on the main criteria, thus meeting the admission requirements.
  iii. Participants in this cluster are categorized as “Accepted.”

The results of this study show that the K-Means Clustering algorithm is an effective tool for grouping job applicants based on three main competencies: AutoCAD Drawing, Supervision Planning Reports, and Adaptability. This clustering produces three categories: “Rejected,” “Considered,” and “Accepted.” Applicants with high adaptability scores tend to fall into the ‘Considered’ or “Accepted” categories, which is in line with the findings of Brown & Hesketh (2005), who emphasize the importance of adaptability in supporting successful career transitions. Groups with strong technical skills in AutoCAD drawing and planning reports also show a higher tendency to be accepted, in line with the needs of the modern skills-based labor market.

Cluster visualization through 2D and 3D scatter plots provides intuitive insights into the distribution patterns of applicants based on their main characteristics. This graph shows that applicants with high scores on technical and adaptability variables are more likely to be in the “Accepted” cluster. As explained by Zhang et al. (2024), data visualization allows for a deeper understanding of the relationships between variables, thereby supporting evidence-based decision making. Additionally, the distribution in this graph provides a clear separation between clusters, allowing companies to focus on the group of applicants with the greatest potential. The “Under Consideration” cluster offers a unique opportunity for companies to identify candidates with great potential who require further evaluation or additional training. As highlighted by Rawat et al. (2024), targeted training can improve applicants’ abilities, ensuring that they can meet company standards in the long term. Thus, this approach not only helps companies understand the technical strengths of applicants but also provides insight into how these individuals can sustainably contribute to the organization, as also explained by Hurbean, et al. (2023).

The data-driven approach applied in this study aligns with the views of Akkermans et al. (2024), who emphasize that data-driven analysis provides a more objective and strategic basis for decision-making in the field of human resources. By basing decisions on clustering data, companies can improve the efficiency of the selection process and focus on the most suitable candidates to meet the needs of construction projects.

Overall, this study confirms that technical and adaptive abilities evaluated through clustering contribute significantly to individual and organizational success. As stated by Donald et al. (2024), this approach not only identifies applicants’ strengths but also provides strategic guidance on how to integrate those skills into organizational needs. Therefore, this clustering is relevant not only for the selection process but also for data-driven workforce development.

## 4. Conclusions

The results of this study confirm that the K-Means Clustering algorithm is an effective tool for grouping job applicants at construction consulting firms based on three key competencies: AutoCAD Drawing, Supervision Planning Reports, and Adaptability. These three variables reflect the primary needs of the construction industry, where technical skills and soft skills are important indicators in determining the suitability of applicants.

The clustering process resulted in three groups: “Rejected,” “Considered,” and “Accepted.” The “Rejected” cluster includes applicants with overall scores below the expected standard, indicating significant deficiencies in one or more of the core competencies evaluated. The “Under Consideration” cluster identifies applicants with average scores, indicating potential but requiring further evaluation or additional training to meet professional standards. Meanwhile, the “Accepted” cluster includes applicants with the highest scores, indicating readiness for immediate placement in a professional work environment, as they have fully met competency expectations.

This clustering process has direct implications for decision-making in recruitment. By understanding the distribution of applicants across these three clusters, management can focus more on candidates with the best potential to contribute effectively to construction projects. Additionally, visualizing the clustering results in the form of 2D and 3D scatter plots provides deep insights into the relationships between variables, enabling companies to identify priority competencies that need to be improved through targeted training programs.

The application of the K-Means Clustering method to job applicant data offers strategic insights into the workforce selection process. These results align with Diván’s (2017) perspective that data-driven analysis can strengthen the recruitment process, particularly in identifying groups of individuals most suited to the company’s needs. Additionally, as emphasized by Rawat et al. (2024), structured training programs can play a crucial role in enhancing applicants’ capabilities within the “Considered” cluster, ensuring their alignment with the organization’s long-term needs. These findings also reflect the importance of adaptability in facing job insecurity, as outlined by Van der Heijden et al. (2024), who show that this ability contributes significantly to career sustainability and reduced labor instability.

Although this clustering approach has proven effective in identifying potential applicants, its success is highly dependent on the accuracy and completeness of the data used. Further research could explore additional variables or integrate other clustering algorithms to improve the categorization process.

The unique contribution of this research lies in the application of K-Means Clustering to job applicant data in the construction sector—an industry that heavily relies on a balance between technical skills and adaptability. This focused approach provides a framework that can be adapted by other sectors requiring alignment between recruitment processes and specific competency needs. Beyond its technical contributions, this research introduces a data-driven approach that enables companies to enhance hiring accuracy, selection efficiency, and the development of competencies aligned with industry needs. As highlighted by Donald et al. (2024) and Akkermans et al. (2024), a data-driven approach provides a solid foundation for aligning individual potential with organizational goals, supporting both short-term recruitment needs and long-term sustainable workforce management. Therefore, this approach not only addresses challenges in evaluating applicants but also builds a foundation for sustainable talent management in the construction industry.

## Ethical approval

Ethical review and approval were not required for this study because the researchers analyzed fully anonymized secondary data that had been lawfully transferred by CV Ardantama Putra Perkasa under a formal Data Usage Agreement (No. 12/X/S-K/APP/2024). According to Indonesian national research ethics regulations (Permenkes RI No. 74/2016, Article 11) and the general principles of the Declaration of Helsinki, research involving secondary anonymized non-clinical data that cannot identify individuals is exempt from institutional ethical review. Therefore, this study qualifies for an ethics exemption.

## Informed consent

Informed consent for data use was not obtained directly by the researchers, as all data were collected by CV Ardantama Putra Perkasa under standard recruitment procedures.

The company confirmed, through the Data Usage Agreement (No. 12/X/S-K/APP/2024), that job applicants had authorized the use of their anonymized recruitment test results for evaluation and administrative purposes in accordance with Indonesian data protection regulations (UU ITE and PP 71/2019). Because the researchers received only anonymized secondary data and had no access to identifiable information, this study meets the criteria for consent exemption.

## Clinical trial registration

Not applicable.

## Data Availability

The anonymized job applicant dataset is not publicly available due to confidentiality agreements with CV Ardantama Putra Perkasa. Researchers may request access by contacting the corresponding author at danieljesayanto.2023@student.uny.ac.id. Access may be granted for legitimate academic research and is subject to:
a. signing a Data Use Agreement; b. agreeing not to attempt re-identification; c. compliance with Indonesian data protection regulations (UU ITE and PP 71/2019); d. approval from CV Ardantama Putra Perkasa as the data owner. Supplementary figures, R scripts, and documentation are openly available in Zenodo at **https://doi.org/10.5281/zenodo.17736891** ( Jaya, 2025) under the Creative Commons Attribution 4.0 International (CC BY 4.0) license.

## References

[ref1] Akkermans J Donald WE Jackson D : Are we talking about the same thing? The case for stronger connections between graduate and worker employability research. *Career Dev. Int.* 2024;29(1):80–92. 10.1108/CDI-08-2023-0278

[ref2] Agustina N Prihandoko P : Perbandingan algoritma K-Means dengan Fuzzy C-Means untuk clustering tingkat kedisiplinan kinerja karyawan. *Jurnal RESTI (Rekayasa Sistem dan Teknologi Informasi).* 2018;2(3):621–626. 10.29207/resti.v2i3.492

[ref3] Brown P Hesketh A : The mismanagement of talent: Employability and jobs in the knowledge economy. *Ind. Labor Relat. Rev.* 2005. 10.2189/asqu.2005.50.2.306

[ref4] Chen Yu : *K-Means clustering.* Indiana University;2020.

[ref5] Darmi Y Setiawan A : Penerapan metode clustering K-Means dalam pengelompokan penjualan produk. *Jurnal Media Infotama.* 2016;12(2):148–157.

[ref6] Diván M : Data-driven decision making. *2017 IEEE International Conference on Technological Innovations in ICT for Agriculture and Rural Development (TIAR).* IEEE;2017; pp.50–56. 10.1109/ICTUS.2017.8285973

[ref7] Donald WE Van der Heijden BIJM Manville G : (Re) Framing sustainable careers: Toward a conceptual model and future research agenda. *Career Dev. Int.* 2024;29(5):513–526. 10.1108/CDI-02-2024-0073

[ref8] El Achmar D Bhagat R : The conceptual relation between human resource management (HRM) and competency mapping. *International Journal of Teaching & Education.* 2023.

[ref9] Fadhli M : Manajemen peningkatan mutu pendidikan. *Tadbir: Jurnal Studi Manajemen Pendidikan.* 2017;1(2):215–240. 10.29240/jsmp.v1i2.295

[ref10] Firza F Sarjono S : Penerapan algoritma K-Means dalam metode clustering untuk peminatan jurusan bagi siswa Swasta Pelita Raya Kota Jambi. *Jurnal Manajemen Sistem Informasi.* 2020;5(3):371–382.

[ref11] Gangl M : Labor market structure and re-employment rates: Unemployment dynamics in West Germany and the United States. *Research in Social Stratification and Mobility.* 2003;20:185–224. 10.1016/S0276-5624(03)20004-4

[ref12] Gie W Jollyta D : Perbandingan Euclidean dan Manhattan untuk optimasi cluster menggunakan Davies-Bouldin Index: Status COVID-19 wilayah Riau. *Prosiding Seminar Nasional Riset Information Science (SENARIS).* 2020, July;2:187–191.

[ref13] Green AE Hoyos M Li Y : *Job search study: Literature review and analysis of the Labour Force Survey.* London: Department for Work and Pensions;2011.

[ref14] Hurbean L Miliaru F Muntean M : The impact of business intelligence and analytics adoption on decision-making effectiveness and managerial work performance. *Scientific Annals of Economics and Business.* 2023;70:43–54. 10.47743/saeb-2023-0012

[ref15] Irmawan I Sagharmata FA Ruthriana F : Analisis dampak pembangunan Kota Hutan (Forest City) (Studi kasus: Ibu Kota Nusantara (IKN), Kalimantan). *Prosiding Seminar Rekayasa Teknologi (SemResTek).* 2023;299–304.

[ref16] Jain AK Murty MN Flynn PJ : Data clustering: A review. *ACM Computing Surveys (CSUR).* 1999;31(3):264–323. 10.1145/331499.331504

[ref17] Jaya DJ : Supplementary Materials for “Application of K-Means Clustering for Job Applicant Analysis in Construction Firms Using R”.[Data set]. *Zenodo.* 2025. 10.5281/zenodo.17736891 PMC13294664 42368335

[ref18] Kassambara A : *Practical guide to cluster analysis in R.* STHDA; 1st ed. 2017. Reference Source

[ref19] London HH : *Principles and techniques of vocational guidance.* Ohio: Charles E. Merrill Publishing Company;1973.

[ref20] Manikandan S Caroline AL Kanniamma D : The study on clustering analysis in data mining. *International Journal of Data Mining Techniques and Applications.* 2018;7(1):46–49.

[ref21] Pala SK : Use and applications of data analytics in human resource management and talent acquisition. *International Journal of Enhanced Research in Science, Technology & Engineering.* 2021;10:2319–7463.

[ref22] Purba W Tamba S Saragih J : The effect of mining data K-Means clustering toward students profile model drop out potential. *IOP Conference Series: Journal of Physics.* 2018;1007(1):012046–012049. 10.1088/1742-6596/1007/1/012049

[ref23] Rawat A Nadavulakere S Isenhour L : Career enhancement strategies, supportive work relationships and subjective career success: The moderating role of family–work conflict. *Career Dev. Int.* 2024;29(4):421–433. 10.1108/CDI-06-2023-0160

[ref24] Smith SC Todaro MP : *Economic development.* Boston: Pearson Education; 12th ed. 2015.

[ref25] Supriyanti SS Kusmayanti JD Paluseri ARA : Pemberdayaan masyarakat sekitar di wilayah Ibu Kota Nusantara. *Masyarakat Indonesia.* 2023;49(1):93–102.

[ref26] Van der Heijden BIJM Hofer A Semeijn J : “Don’t you worry’bout a thing” – The moderating role of age in the relationship between qualitative job insecurity and career sustainability. *Career Dev. Int.* 2024;29(5):527–543. 10.1108/CDI-08-2023-0280

[ref27] Widiyaningtyas T Prabowo MIW Pratama MAM : Implementation of K-Means clustering to distribution of high school teachers. *Proceeding EECSI, Yogyakarta, 19–21 September.* 2017, September;49–54.

[ref28] Widodo SE : *Manajemen pengembangan sumber daya manusia.* Yogyakarta: Pustaka Pelajar;2018.

[ref29] Wiharto W Suryani E : The comparison of clustering algorithms K-Means and Fuzzy C-Means for segmentation retinal blood vessels. *Acta Informatica Medica.* 2020;28(1):42–46. 10.5455/aim.2020.28.42-47 32210514 PMC7085333

[ref30] Zhang M Zhou S Wu Y : Pressure from social media: Influence of social media usage on career exploration. *Career Dev. Int.* 2024;29(1):93–112. 10.1108/CDI-01-2023-0016

