# Application of K-Means Clustering for Job Applicant Analysis in Construction Firms Using R

**DOI:** 10.12688/f1000research.172383.2

**Published:** 2026-03-12

**Authors:** Daniel Jesayanto Jaya, Wahyu Muhammad Ramdhani, Endang Wati, Yogi Novario Nandes, Ilma Zahriyatun Nadhiroh, Reza Bakhrun Fidianto Pade

**Affiliations:** 1 Technology and Vocational Education and Training, Universitas Negeri Yogyakarta, Yogyakarta, Special Region of Yogyakarta, 55282, Indonesia; 2 Building Engineering Education, Universitas Negeri Jakarta, East Jakarta, Special Capital Region of Jakarta, Indonesia; 3 Educational Research and Evaluation, Universitas Negeri Yogyakarta, Yogyakarta, Special Region of Yogyakarta, 55282, Indonesia; 4 English Language Education, Universitas Negeri Yogyakarta, Yogyakarta, Special Region of Yogyakarta, 55282, Indonesia

**Keywords:** K-Means Clustering; data-driven recruitment; workforce selection; cluster visualization; construction competencies

## Abstract

This study applies K-Means clustering to segment job applicant test data from a construction consulting firm to support data-driven screening decisions. From 161 applicants, 30 candidates who met the document-screening requirements were invited for in-person testing and included in the analysis. Three assessment variables were used: AutoCAD drafting skills, planning and supervision report-writing skills, and adaptability. Using R, K-Means clustering was performed to partition candidates into three groups based on multivariate similarity patterns, and the resulting group structure was visualized using 2D and 3D scatter plots. The clustering output revealed distinct competency profiles: one group characterized by generally lower scores across the three variables, a second group with moderate and mixed scores, and a third group with consistently higher scores. Internal validity indices suggested modest separation (mean silhouette = 0.16; Davies–Bouldin Index = 2.05), consistent with exploratory clustering on a small pre-screened sample. These patterns provide a structured interpretation of applicant diversity and can inform practical recruitment actions such as prioritizing candidates for interviews, identifying borderline profiles for additional evaluation, and designing targeted upskilling recommendations for specific competency gaps. Overall, this study demonstrates how unsupervised clustering of routine recruitment test results can enhance transparency and consistency in early-stage applicant evaluation within construction-sector hiring.

## 1. Introduction

### 1.1 Research background

In the modern workplace, workforce selection is a critical component of human resource development, particularly in sectors that require a combination of technical expertise and adaptive capability. Career development and career transformation are influenced not only by formal qualifications but also by individuals’ ability to adapt to changing work environments and collaborate effectively with diverse stakeholders. Data-driven approaches to workforce analysis have therefore gained attention as tools to support more structured and transparent evaluation processes ( Pala, 2021).

Recruitment involves more than sourcing candidates; it requires systematic decision-making informed by job analysis, organizational needs, and available labor characteristics ( Widodo, 2018). Job analysis plays a central role in defining task requirements, competency expectations, and qualification standards, thereby helping organizations align applicants with role-specific demands. From the applicant’s perspective, successful job search outcomes depend on understanding personal competencies, evaluating labor market opportunities, and developing skills that match employer expectations ( London, 1973).

In the construction sector, technical competencies such as AutoCAD drafting, the ability to prepare planning and supervision reports, and adaptability to dynamic project environments are particularly valued ( Gangl, 2003). These competencies are increasingly important in large-scale infrastructure development contexts. In Indonesia, national strategic projects such as the Nusantara Capital City (Ibu Kota Nusantara, IKN) development have intensified demand for construction personnel with both technical proficiency and social adaptability ( Irmawan et al., 2023; Supriyanti et al., 2023). Managing and interpreting recruitment assessment data in such contexts presents practical challenges, especially when organizations must evaluate multiple competency dimensions simultaneously.

Cluster analysis offers a data-driven approach to explore patterns within applicant assessment data by grouping individuals with similar characteristics. Clustering techniques partition data into internally homogeneous and externally heterogeneous groups, thereby supporting structured interpretation of complex multivariate information ( Jain et al., 1999). Among these techniques, K-Means clustering is widely used due to its computational simplicity and interpretability, making it suitable for exploratory analysis of recruitment-related datasets. In recruitment contexts, clustering can be applied to post-screening assessment data to identify competency profiles rather than to make automated hiring decisions.

Beyond operational efficiency, the use of data-driven tools in recruitment raises broader issues of transparency, governance, and fairness in algorithm-assisted selection. International guidance emphasizes that AI-enabled assessment should be accompanied by risk management, documentation, and ongoing monitoring of unintended impacts ( NIST, 2023). In addition, U.S. Equal Employment Opportunity Commission (EEOC) guidance highlights that employers should assess whether algorithmic or AI-based selection procedures produce adverse impact under Title VII and aligns such assessment with the Uniform Guidelines on Employee Selection Procedures ( EEOC, 2023). Similarly, the European Union Artificial Intelligence Act classifies certain AI systems used in employment-related contexts as high-risk, reinforcing expectations for accountability and safeguards when analytics influence employment decisions ( European Union, 2024). Accordingly, this study positions K-Means clustering as an exploratory decision-support technique rather than an automated hiring system; cluster labels are interpreted cautiously as descriptive competency profiles and are intended to complement human review rather than replace managerial judgment.

This study applies K-Means clustering to recruitment test data from a construction consulting firm, focusing on candidates who passed document screening and completed in-person assessments. Using three core variables—AutoCAD drafting skills, planning and supervision report-writing skills, and adaptability—the study demonstrates how unsupervised clustering can support exploratory analysis of applicant competency profiles within a real organizational context.

### 1.2 Literature review

Clustering is an unsupervised analytical technique used to group objects into clusters based on attribute similarity, such that objects within the same cluster exhibit higher similarity than those in other clusters ( Jain et al., 1999). By minimizing within-cluster variation and maximizing between-cluster differences, clustering supports pattern discovery and interpretation in complex datasets ( Manikandan et al., 2018; Darmi & Setiawan, 2016). For organizational and workforce analytics, clustering provides a data-driven means of understanding heterogeneity among individuals without requiring predefined class labels.

Among various clustering approaches, K-Means clustering is one of the most widely applied methods due to its simplicity, efficiency, and interpretability. K-Means partitions data into *k* clusters by iteratively assigning observations to the nearest centroid and updating centroid positions until convergence is achieved ( Jain et al., 1999). Because of its relatively low computational cost, K-Means is suitable for applied settings where rapid analysis and transparent interpretation are required ( Fadhli, 2017).

Previous studies demonstrate applicability across domains. In educational research, K-Means has been used to analyze student preferences and learning achievement patterns ( Firza & Sarjono, 2020). In organizational contexts, it has been applied to group employees based on discipline and performance indicators to support human resource decision-making ( Agustina & Prihandoko, 2018). Comparative studies suggest that while alternatives such as Fuzzy C-Means may offer advantages in some conditions, K-Means remains computationally efficient and practical for many real-world applications ( Wiharto & Suryani, 2020).

1.2.1 K-Means algorithm

K-Means is a partition-based clustering algorithm that divides data into a predefined number of clusters by minimizing the average distance between data points and their respective cluster centroids ( Widiyaningtyas et al., 2017). The algorithm operates iteratively, beginning with the selection of initial centroid values and proceeding through repeated reassignment of data points based on distance calculations until cluster membership stabilizes ( Purba et al., 2018). Prior work emphasizes that K-Means can be sensitive to initialization and the scale of input variables, highlighting the need for transparent methodological choices in applied studies ( Jain et al., 1999).

1.2.2 Worker recruitment

Recruitment is a strategic organizational process aimed at attracting and selecting individuals whose competencies align with job requirements and organizational objectives. Job analysis plays a critical role in defining tasks, responsibilities, and qualification standards, thereby guiding recruitment and selection decisions ( Widodo, 2018). In the construction sector, recruitment emphasizes a combination of technical competencies—such as drafting and report preparation—and adaptive capabilities, reflecting the dynamic and collaborative nature of construction projects ( Gangl, 2003). The job search process seeks to match job seekers with appropriate opportunities and can be supported through technology-enabled and data-driven methods ( Green et al., 2011). Given the multidimensionality of applicant data, clustering methods such as K-Means offer a way to organize assessment results into interpretable competency profiles that can support early-stage evaluation ( Jain et al., 1999).

## 2. Methods

### 2.1 Research Design

This study employed a quantitative, exploratory research design using unsupervised clustering to analyze recruitment assessment data from a construction consulting firm. The primary objective was to explore competency-based grouping patterns among job applicants using K-Means clustering as a decision-support tool, rather than to predict hiring outcomes or evaluate post-employment performance.

**Figure 1.**
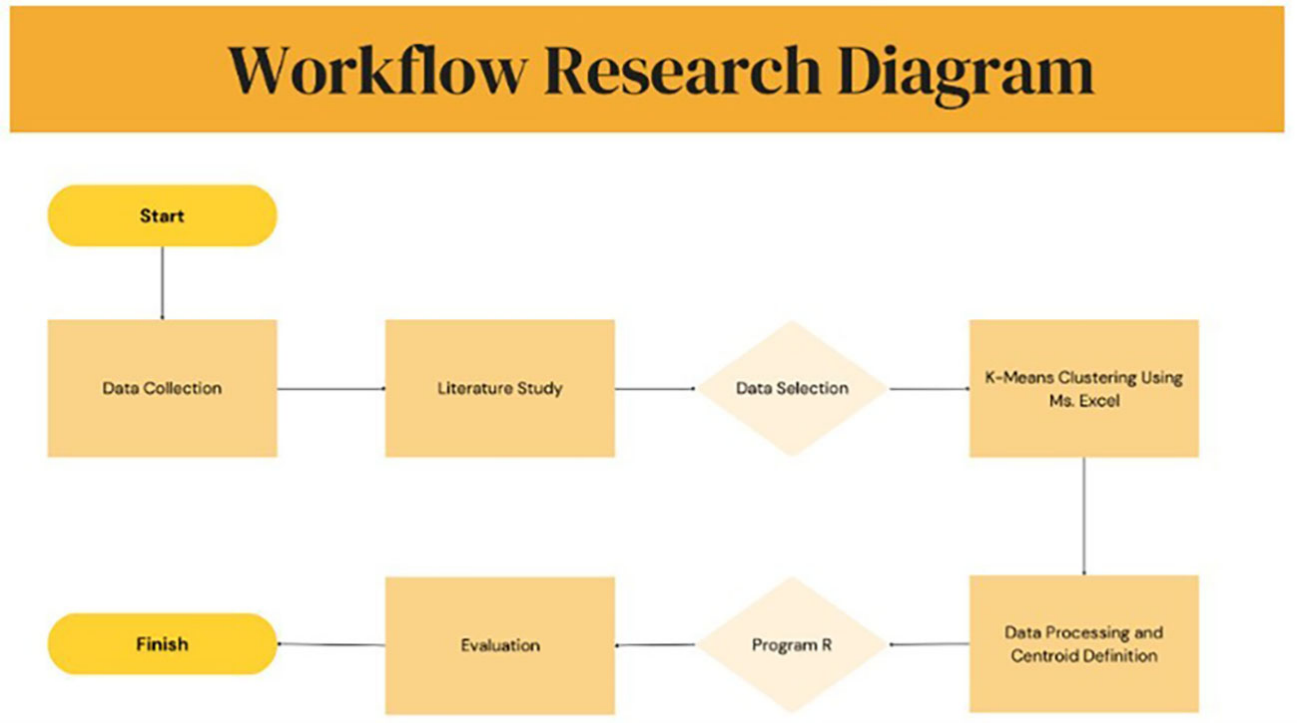
Workflow research diagram.

### 2.2 Data source and participant selection

The data were obtained from CV Ardantama Putra Perkasa as part of its internal recruitment process. Although the vacancy was advertised through JobStreet Indonesia, all data analyzed in this study originated exclusively from the company’s internal screening and testing procedures.

A total of 161 applicants applied for the position. Applicants were shortlisted through the company’s standard document-screening procedure conducted by the HR team and the hiring unit. Screening focused on administrative completeness and role relevance, including:

(i) completeness of required documents;

(ii) educational background and relevance to construction consulting work;

(iii) evidence of relevant technical exposure (e.g., drafting/reporting-related tasks or portfolio where available); and

(iv) basic eligibility criteria specified in the vacancy announcement.

From this screening stage, 30 candidates who met the minimum requirements were invited for in-person testing. Only these 30 candidates were included in the clustering analysis because complete assessment scores were available for all three variables. This design improves internal consistency of the tested dataset but limits generalizability to the full applicant pool.

### 2.3 Assessment variables

Candidates were evaluated using three competency indicators relevant to construction consulting roles:

1. AutoCAD drafting skills;
2. planning and supervision report-writing skills;
3. adaptability.

Each variable was assessed on a numerical scale from 0 to 100, with higher scores indicating stronger performance.

### 2.4 Data preprocessing, outlier, and sensitivity checks

The dataset was reviewed for completeness and consistency. All 30 candidates had complete scores across the three assessment variables; therefore, no records were excluded at this stage. Because all variables were measured using the same scale (0–100), the analysis used raw scores without additional normalization to preserve the meaning of the original assessment scores.

A basic outlier and sensitivity check was conducted by examining distances to cluster centroids and visually inspecting the 3D scatter plot. A leave-one-out sensitivity test removing the most distant observation did not materially change the overall three-cluster interpretation; validity metrics changed only slightly (mean silhouette increased from 0.16 to approximately 0.18; DBI remained approximately 2.0). This suggests the reported structure is not driven by a single extreme case.

### 2.5 Clustering procedure

K-Means clustering was applied to group candidates based on similarity across the three assessment variables. The number of clusters was set to *k* = 3, reflecting the company’s practical need to differentiate candidates into three evaluative groups for recruitment support.

Initial centroid values were specified as starting points based on preliminary inspection of score distributions during exploratory analysis. These initial values were used to initiate iteration rather than to impose predetermined outcome categories. Euclidean distance was used to assign candidates to the nearest centroid, after which centroid positions were updated as the mean of cluster members. The algorithm iterated until cluster assignments stabilized.

The clustering workflow was implemented using a combination of spreadsheet-based calculations (for transparency of manual steps) and the R programming language (for reproducibility, validity checks, and visualization). Intermediate iteration tables are provided as extended data.

**2.5.1 Initialization and stability checks**

Because K-Means can be sensitive to initialization, the analysis was repeated in R using the built-in kmeans() function with multiple random initializations (e.g., nstart = 50). Solution stability was assessed by comparing convergence outcomes (within-cluster sum of squares) and checking consistency of cluster memberships across repeated initializations. This step ensured that the reported three-cluster structure was not an artifact of a single starting configuration. Minor membership differences across runs occurred for borderline profiles, which is plausible in small samples with overlapping competency distributions.

### 2.6 Visualization and interpretation

Clustering results were visualized using two-dimensional and three-dimensional scatter plots. Two-dimensional plots illustrated relationships between AutoCAD drafting skills and planning/supervision report-writing skills, while three-dimensional plots incorporated adaptability as a third axis.

Clusters were subsequently labeled as “Rejected,” “Under Consideration,” and “Accepted” based on their relative position in the multivariate competency space. These labels represent analytical interpretations of score patterns and do not constitute formal hiring decisions made by the company.

### 2.7 Scope and methodological limitations

This study focuses on exploratory grouping of recruitment assessment data from a pre-screened subset of applicants. The clustering results were not validated against final hiring decisions or post-employment performance outcomes. Accordingly, findings should be interpreted as structured analytical support rather than definitive evidence of selection effectiveness.

### 2.8 Cluster validity assessment

To provide quantitative support for the cluster structure, internal validity indices were calculated. The silhouette coefficient was computed using Euclidean distances to estimate how well each candidate matched its assigned cluster relative to other clusters. The Davies–Bouldin Index (DBI) was calculated to evaluate average cluster similarity based on within-cluster dispersion relative to between-cluster centroid distances. These indices were interpreted as descriptive diagnostics of separation quality rather than evidence of predictive utility.

## 3. Results and discussion

### 3.1 Applicant characteristics

This study analysed recruitment assessment records from CV Ardantama Putra Perkasa, obtained from the company’s internal testing and selection process. A total of 161 applicants submitted applications, of whom 30 candidates meeting minimum screening criteria were invited for in-person testing. Each candidate was assessed on three indicators measured on a 0–100 scale: AutoCAD drafting skills (X), planning and supervision report-writing skills (Y), and adaptability (Z). Candidate characteristics and scores are summarised in Table 1.

**Table 1.** Applicant demographic data.

Respondent code	Gender	AutoCAD drawing skills (X)	Ability to prepare planning and monitoring reports (Y)	Adaptability (Z)
Resp1	Female	92	75	68
Resp2	Male	68	65	66
Resp3	Male	73	86	87
Resp4	Male	69	74	73
Resp5	Male	78	72	91
Resp6	Female	84	90	92
Resp7	Male	69	76	87
Resp8	Female	95	73	76
Resp9	Female	90	80	85
Resp10	Male	68	82	68
Resp11	Male	63	75	71
Resp12	Male	75	93	77
Resp13	Female	62	72	68
Resp14	Male	90	61	72
Resp15	Female	84	63	90
Resp16	Female	94	70	89
Resp17	Female	73	87	80
Resp18	Female	71	73	95
Resp19	Female	93	62	70
Resp20	Male	90	68	89
Resp21	Female	87	94	87
Resp22	Male	60	90	64
Resp23	Female	65	64	93
Resp24	Male	69	84	75
Resp25	Male	66	63	72
Resp26	Male	95	85	93
Resp27	Male	75	80	83
Resp28	Male	92	85	93
Resp29	Male	71	71	85
Resp30	Male	92	61	88

Overall, the score distribution shows meaningful heterogeneity across candidates—particularly in adaptability and planning/supervision report-writing—indicating variation in both technical and interpersonal readiness. This variability provides a suitable basis for exploratory clustering analysis.

### 3.2 K-Means clustering results

Using K-Means clustering with *k* = 3, the 30 assessed candidates were grouped into three distinct clusters based on similarity across AutoCAD drafting skills, planning and supervision report-writing skills, and adaptability. The final cluster assignments are summarized in Table 2. These clusters represent analytical competency profiles derived from multivariate similarity patterns rather than formal hiring decisions determined by company policy.

**Table 2.** Final clustering results.

Respondent data				Rejected			Under consideration			Accepted			Clustering
				C1(x _1_,y _1_,z _1_)			C2(x _2_,y _2_,z _2_)			C3(x _3_,y _3_,z _3_)			
Name	MA	LPP	KA	67,30	78,50	71,40	73,25	73,13	88,88	91,17	75,33	83,50	
Resp2	68	65	66	14,56			24,84			30,82			Cluster 1 (Rejected)
Resp4	69	74	73	5,07			16,46			24,56			
Resp10	68	82	68	4,93			23,28			28,66			
Resp11	63	75	71	5,56			20,69			30,82			
Resp13	62	72	68	9,05			23,74			33,20			
Resp22	60	90	64	15,50			32,85			39,58			
Resp24	69	84	75	6,79			18,13			25,27			
Resp25	66	63	72	15,57			20,97			30,29			
Resp17	73	87	80	13,37			16,47			21,87			
Resp12	75	93	77	17,35			23,22			24,81			
Resp7	69	76	87	15,89			5,46			22,45			Cluster 2 (Under Consideration)
Resp23	65	64	93	26,12			12,97			30,06			
Resp29	71	71	85	15,97			4,96			20,68			
Resp5	78	72	91	23,26			5,32			15,52			
Resp18	71	73	95	24,51			6,53			23,33			
Resp3	73	86	87	18,22			13,01			21,36			
Resp15	84	63	90	29,41			14,81			15,68			
Resp27	75	80	83	14,00			9,21			16,83			
Resp14	90	61	72	28,67			26,69			18,41			Cluster 3 (Accepted)
Resp19	93	62	70	30,57			29,50			19,06			
Resp1	92	75	68	25,18			28,12			15,53			
Resp6	84	90	92	28,91			20,25			18,40			
Resp8	95	73	76	28,61			25,28			8,74			
Resp9	90	80	85	26,50			18,52			5,04			
Resp16	94	70	89	33,09			20,98			8,17			
Resp20	90	68	89	30,58			17,52			9,24			
Resp21	87	94	87	29,52			25,07			19,44			
Resp26	95	85	93	35,72			25,12			14,09			
Resp28	92	85	93	33,45			22,57			13,58			
Resp30	92	61	88	34,52			22,35			15,05			

The first cluster is characterized by relatively lower combined scores across the three assessed competencies. The second cluster consists of candidates with moderate and mixed competency scores, reflecting intermediate profiles that may warrant further evaluation. The third cluster comprises candidates with consistently higher scores across technical and adaptive dimensions, indicating stronger and more balanced competency profiles.

The clustering process involved iterative centroid updates until cluster memberships stabilized. To maintain readability, detailed iteration tables are provided as extended data, while the main text focuses on the stabilized results and their interpretation. Re-running clustering with multiple random initializations in R produced highly similar solutions, suggesting the three-cluster structure was not dependent on a single manual initialization. Minor membership differences across runs occurred for borderline profiles, which is expected in small samples with partially overlapping competency distributions.

The final clustering output generated from the R environment, including cluster labels and competency scores for each applicant, is presented in Table 3.

**Table 3.** R-generated data table.

No	AutoCAD_Drafting	Planning_Supervision_ Reports	Adaptability	Cluster	Category
1	68	65	66	1	Rejected
2	69	74	73	1	Rejected
3	68	82	68	1	Rejected
4	63	75	71	1	Rejected
5	62	72	68	1	Rejected
6	60	90	64	1	Rejected
7	69	84	75	2	Under Consideration
8	66	63	72	1	Rejected
9	73	87	80	2	Under Consideration
10	75	93	77	2	Under Consideration
11	69	76	87	3	Accepted
12	65	64	93	1	Rejected
13	71	71	85	2	Under Consideration
14	78	72	91	3	Accepted
15	71	73	95	2	Under Consideration
16	73	86	87	2	Under Consideration
17	84	63	90	3	Accepted
18	75	80	83	2	Under Consideration
19	90	61	72	2	Under Consideration
20	93	62	70	2	Under Consideration
21	92	75	68	2	Under Consideration
22	84	90	92	3	Accepted
23	95	73	76	3	Accepted
24	90	80	85	3	Accepted
25	94	70	89	3	Accepted
26	90	68	94	3	Accepted
27	87	84	87	3	Accepted
28	95	85	93	3	Accepted
29	92	85	93	3	Accepted
30	92	61	88	2	Under Consideration

**3.2.1 Cluster validity metrics**

Internal validation indicated modest cluster separation. The mean silhouette coefficient was 0.16, suggesting partial overlap among competency profiles, which is plausible given the small pre-screened sample. The Davies–Bouldin Index was 2.05, indicating moderate distinctiveness among the three clusters. These values support interpreting the clusters as exploratory competency groupings rather than sharply separated classes.

### 3.3 Visualization of cluster structure

**Figure 2.**
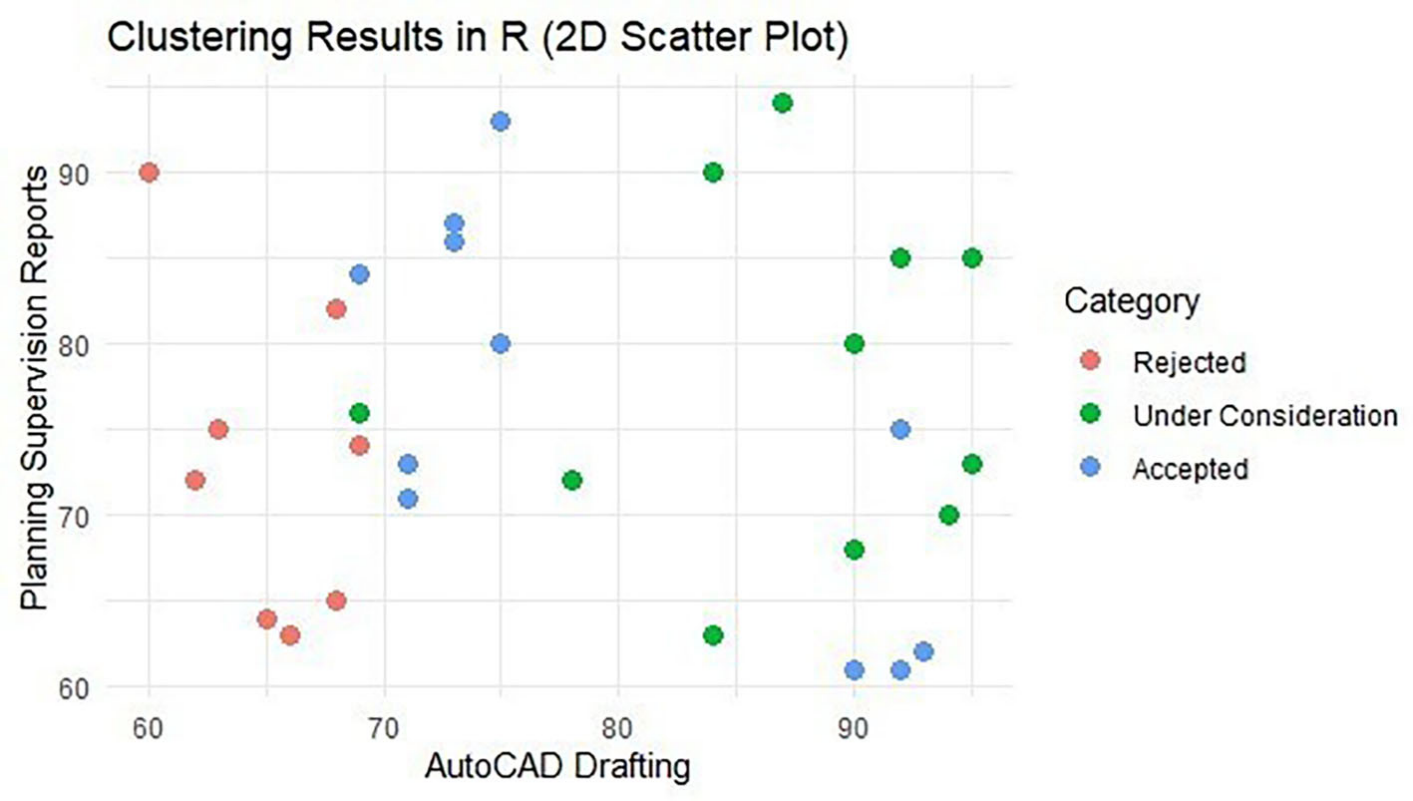
K-means clustering visualization in a 2D scatter plot.

To support interpretation, two-dimensional and three-dimensional visualizations were generated. Figure 2 presents a 2D scatter plot based on AutoCAD drafting skills and planning/supervision report-writing skills, showing visible separation between lower, intermediate, and higher competency profiles along key technical dimensions.

**Figure 3.**
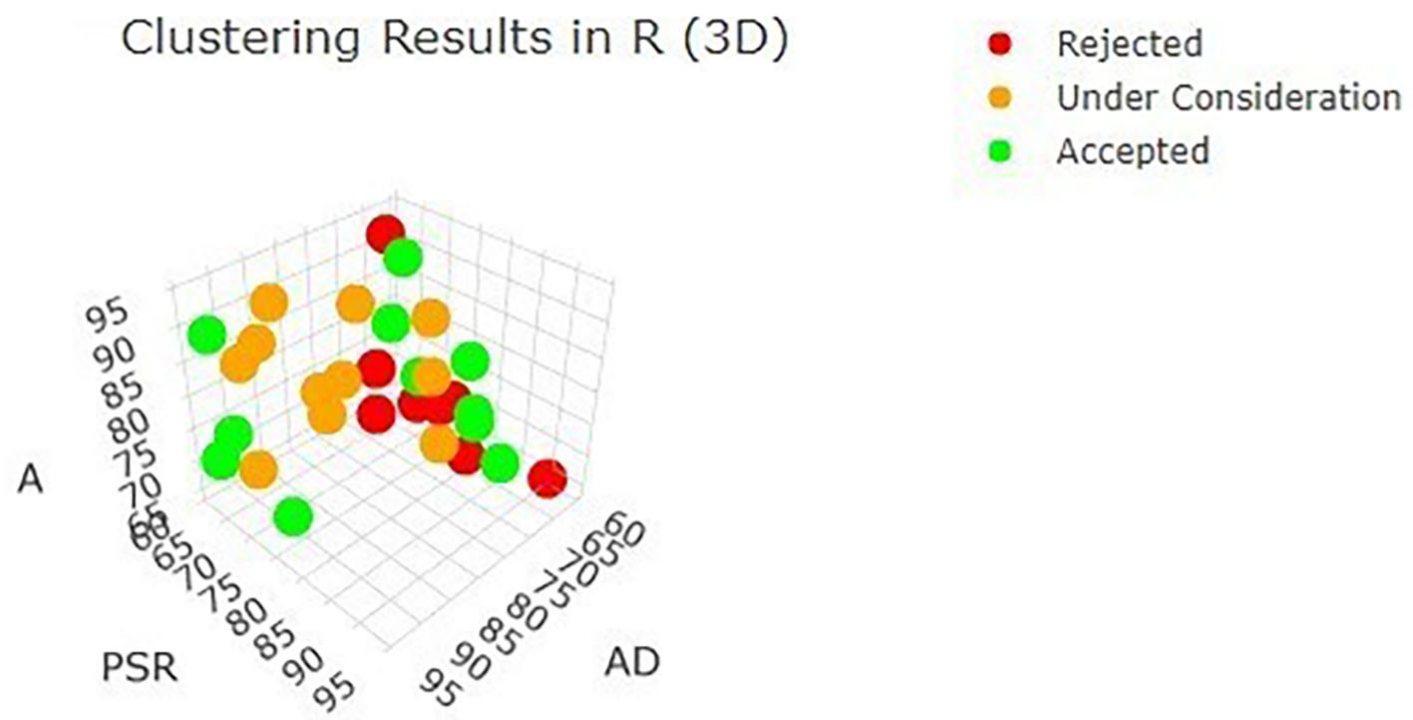
K-means clustering visualization in a 3D scatter plot.

Figure 3 extends the visualization into three dimensions by incorporating adaptability as a third axis. The 3D scatter plot reveals clearer spatial separation among clusters, particularly distinguishing candidates who combine strong technical skills with high adaptability from those with lower overall competency scores.

For clarity of interpretation, the clustered dataset sorted by category is provided in Table 4.

**Table 4.** Sorted dataset by cluster categories.

No	AutoCAD_Drafting	Planning_Supervision_ Report	Adaptability	Cluster	Category
**1**	68	65	66	1	Rejected
**2**	69	74	73	1	Rejected
**3**	68	82	68	1	Rejected
**4**	63	75	71	1	Rejected
**5**	62	72	68	1	Rejected
**6**	60	90	64	1	Rejected
**7**	66	63	72	1	Rejected
**8**	65	64	93	1	Rejected
**9**	69	84	75	2	Under Consideration
**10**	73	87	80	2	Under Consideration
**11**	75	93	77	2	Under Consideration
**12**	71	71	85	2	Under Consideration
**13**	71	73	95	2	Under Consideration
**14**	73	86	87	2	Under Consideration
**15**	75	80	83	2	Under Consideration
**16**	90	61	72	2	Under Consideration
**17**	93	62	70	2	Under Consideration
**18**	92	75	68	2	Under Consideration
**19**	92	61	88	2	Under Consideration
**20**	69	76	87	3	Accepted
**21**	78	72	91	3	Accepted
**22**	84	63	90	3	Accepted
**23**	84	90	92	3	Accepted
**24**	95	73	76	3	Accepted
**25**	90	80	85	3	Accepted
**26**	94	70	89	3	Accepted
**27**	90	68	89	3	Accepted
**28**	87	94	87	3	Accepted
**29**	95	85	93	3	Accepted
**30**	92	85	93	3	Accepted

**Figure 4.**
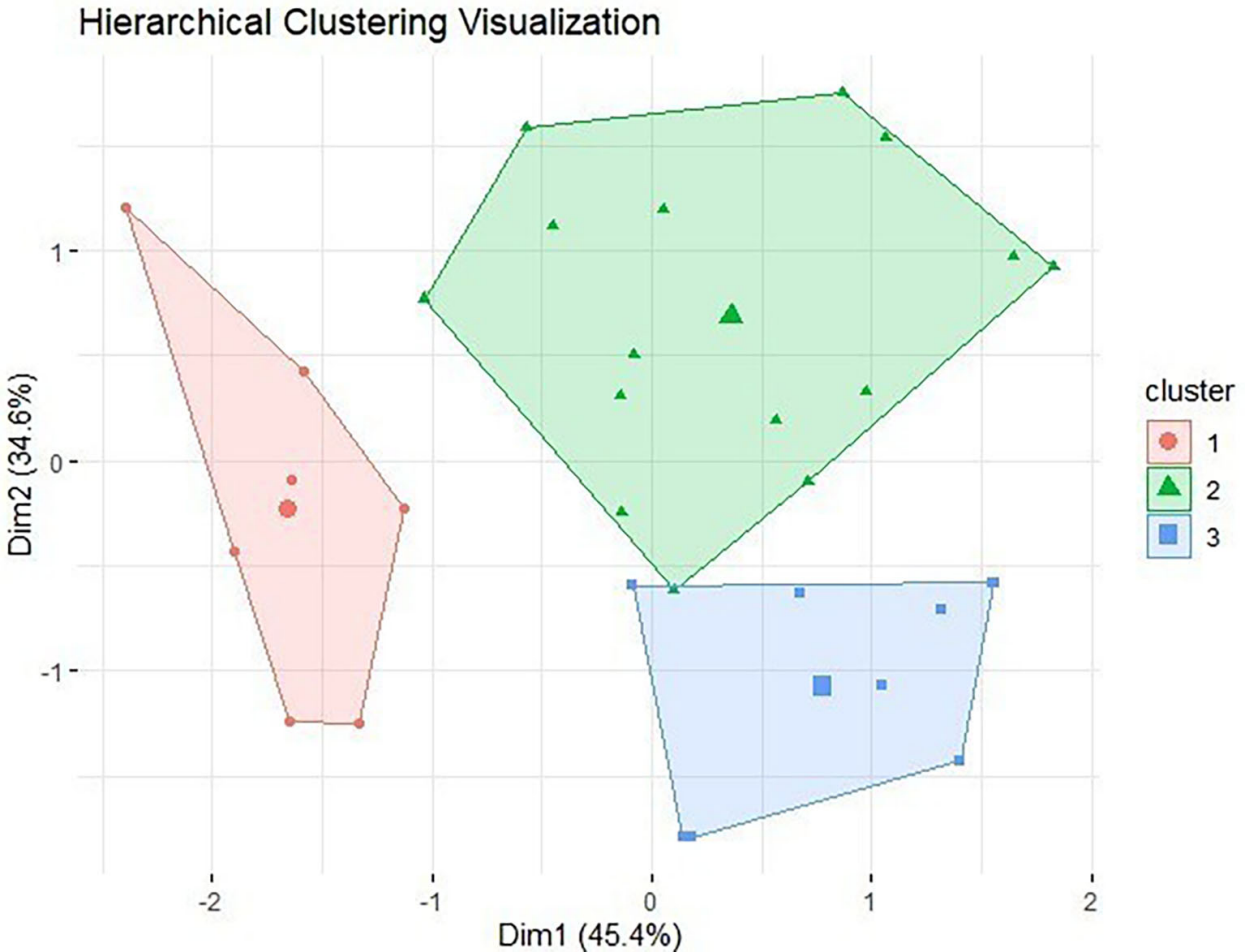
Hierarchical clustering visualization using PCA-projected dimensions.

To further examine structural consistency, hierarchical clustering projected onto principal component space is presented in Figure 4. Although hierarchical clustering was not employed as the primary analytical method, the observed grouping patterns broadly align with the K-Means classification, providing additional support for the stability of the three-cluster structure within this dataset.

### 3.4 Interpretation and discussion

The clustering results demonstrate that K-Means can be used as an exploratory tool to organize recruitment assessment data into interpretable competency profiles within a construction consulting context. Candidates grouped in the higher-scoring cluster tend to exhibit stronger performance across both technical and adaptive dimensions, consistent with prior research emphasizing the importance of combining technical competence with adaptability in project-based and construction-related work environments ( Gangl, 2003; Brown & Hesketh, 2005).

The intermediate cluster represents candidates with mixed strengths, suggesting development potential rather than clear acceptance or rejection outcomes. This aligns with literature highlighting the role of structured training and targeted skill development in enhancing workforce readiness and career progression ( Rawat et al., 2024). Rather than constituting definitive recruitment decisions, this cluster highlights individuals who may benefit from managerial attention, follow-up assessment, or additional training.

Importantly, the clustering approach does not replace professional judgment in recruitment. Instead, it provides a structured analytical perspective that can support transparency and consistency in early-stage evaluation. This aligns with contemporary views that HR analytics is most effective when it complements human expertise rather than automates decision-making processes ( Akkermans et al., 2024).

From an ethical and governance perspective, the analysis is intended to structure early-stage assessment information rather than to automate acceptance decisions. Guidance on trustworthy AI and employment decision tools emphasizes the need for documentation, monitoring, and attention to bias risks when analytics are used in consequential settings ( NIST, 2023; EEOC, 2023). Accordingly, the cluster labels in this study are treated as descriptive competency profiles and should be used alongside human review, transparent documentation, and periodic evaluation of potential disparate impact.

### 3.5 Methodological considerations and limitations

Several limitations should be considered when interpreting these findings. First, the analysis was conducted on a pre-screened subset of candidates who passed document screening and participated in in-person testing; therefore, results may not generalize to the full applicant pool. Second, internal validity indices indicated modest separation, suggesting partially overlapping competency profiles that are plausible in a small filtered sample. Third, the clusters were not externally validated against final hiring decisions, expert HR evaluation, or subsequent job performance outcomes.

Despite these limitations, the results illustrate how clustering can function as a practical decision-support tool in recruitment contexts involving multidimensional competency assessments. Future research could extend this approach by applying clustering to larger and more diverse applicant pools, incorporating additional competency indicators, comparing alternative clustering methods, and validating cluster profiles against post-hire performance indicators.

## 4. Conclusions

This study explored the use of K-Means clustering as an exploratory analytical approach for organizing recruitment assessment data in a construction consulting context, based on three competencies: AutoCAD drafting skills, planning and supervision report-writing skills, and adaptability. Using data from a pre-screened group of applicants, the analysis identified three distinct competency profiles reflecting different patterns of technical and adaptive capabilities.

The identified clusters indicate that applicants with stronger and more balanced combinations of technical competence and adaptability tend to form a distinct group, while candidates with mixed or lower competency profiles are grouped separately. These results should be interpreted as analytical groupings based on similarity patterns rather than as definitive hiring decisions or evidence of predictive effectiveness. This interpretation is consistent with conceptual discussions emphasizing the importance of adaptability and skill alignment in contemporary labor markets ( Gangl, 2003; Brown & Hesketh, 2005).

Quantitative diagnostics suggested modest separation (mean silhouette = 0.16; DBI = 2.05), supporting cautious interpretation of the clusters as exploratory profiles in a small screened sample. The use of two-dimensional and three-dimensional visualizations enhanced interpretability by illustrating how multivariate competency combinations differentiate applicant profiles. The observed alignment between K-Means results and supporting hierarchical visualization further suggests structural consistency within the analyzed dataset, although external validation against hiring outcomes or job performance was beyond the scope of this study.

From a practical standpoint, the findings suggest that clustering-based analysis may support early-stage recruitment evaluation by helping organizations structure and interpret multidimensional assessment data in a transparent and systematic manner. More broadly, clustering as a decision-support mechanism can be situated within wider discussions on data-driven analysis as a means of structuring managerial judgment rather than replacing it ( Diván, 2017). When used alongside professional expertise, such approaches align with contemporary perspectives on human resource analytics that emphasize analytical support over automated decision-making ( Akkermans et al., 2024).

In addition, the presence of an intermediate competency cluster highlights applicants who may benefit from further evaluation or targeted skill development initiatives, echoing research on structured training and career development ( Rawat et al., 2024; Donald et al., 2024). While career sustainability and job insecurity were not directly examined, the inclusion of adaptability as a clustering dimension resonates with broader discussions on adaptive capacity in uncertain career contexts ( Van der Heijden et al., 2024).

Overall, this study provides a practical illustration of how unsupervised clustering techniques can be applied to recruitment assessment data in the construction sector. By emphasizing transparency, interpretability, and cautious use of analytics within governance and fairness considerations ( NIST, 2023; EEOC, 2023), the study contributes to ongoing discussions on data-driven decision-support tools for workforce selection and development.

## Ethical approval

Ethical review and approval were not required for this study because the researchers analyzed fully anonymized secondary data that had been lawfully transferred by CV Ardantama Putra Perkasa under a formal Data Usage Agreement (No. 12/X/S-K/APP/2024). According to Indonesian national research ethics regulations (Permenkes RI No. 74/2016, Article 11) and the general principles of the Declaration of Helsinki, research involving secondary anonymized non-clinical data that cannot identify individuals is exempt from institutional ethical review. Therefore, this study qualifies for an ethics exemption.

## Informed consent

Informed consent for data use was not obtained directly by the researchers, as all data were collected by CV Ardantama Putra Perkasa under standard recruitment procedures. The company confirmed, through the Data Usage Agreement (No. 12/X/S-K/APP/2024), that job applicants had authorized the use of their anonymized recruitment test results for evaluation and administrative purposes in accordance with Indonesian data protection regulations (UU ITE and PP 71/2019). Because the researchers received only anonymized secondary data and had no access to identifiable information, this study meets the criteria for consent exemption.

## Clinical trial registration

Not applicable.

## Data Availability

The anonymized job applicant dataset is not publicly available due to confidentiality agreements with CV Ardantama Putra Perkasa. Access may be granted for legitimate academic research upon reasonable request to the corresponding author ( danieljesayanto.2023@student.uny.ac.id), subject to approval by the data owner and compliance with Indonesian data protection regulations (UU ITE and PP 71/2019), including signing a Data Use Agreement and a commitment not to attempt re-identification. Extended data supporting this study, including R scripts, clustering iteration tables, visualizations, and documentation, are openly available in Zenodo at **https://doi.org/10.5281/zenodo.18501546** ( Jaya, 2026) under the Creative Commons Attribution 4.0 International (CC BY 4.0) license.

## References

[ref1] Akkermans J Donald WE Jackson D : Are we talking about the same thing? The case for stronger connections between graduate and worker employability research. *Career Dev. Int.* 2024;29(1):80–92. 10.1108/CDI-08-2023-0278

[ref2] Agustina N Prihandoko P : Perbandingan algoritma K-Means dengan Fuzzy C-Means untuk clustering tingkat kedisiplinan kinerja karyawan. *Jurnal RESTI (Rekayasa Sistem dan Teknologi Informasi).* 2018;2(3):621–626. 10.29207/resti.v2i3.492

[ref3] Brown P Hesketh A : The mismanagement of talent: Employability and jobs in the knowledge economy. *Ind. Labor Relat. Rev.* 2005. 10.2189/asqu.2005.50.2.306

[ref4] Chen Yu : *K-Means clustering.* Indiana University;2020.

[ref5] Darmi Y Setiawan A : Penerapan metode clustering K-Means dalam pengelompokan penjualan produk. *Jurnal Media Infotama.* 2016;12(2):148–157.

[ref6] Diván M : Data-driven decision making. *2017 IEEE International Conference on Technological Innovations in ICT for Agriculture and Rural Development (TIAR).* IEEE;2017; pp.50–56. 10.1109/ICTUS.2017.8285973

[ref7] Donald WE Van der Heijden BIJM Manville G : (Re) Framing sustainable careers: Toward a conceptual model and future research agenda. *Career Dev. Int.* 2024;29(5):513–526. 10.1108/CDI-02-2024-0073

[ref31] EEOC : *Select Issues: Assessing Adverse Impact in Software, Algorithms, and Artificial Intelligence Used in Employment Selection Procedures Under Title VII of the Civil Rights Act of 1964 (Technical Assistance; **EEOC-NVTA-2023-2**, Issue Date: **2023-05-18**).* U.S. Equal Employment Opportunity Commission;2003. https://data.aclum.org/storage/2025/01/EOCC_www_eeoc_gov_laws_guidance_select-issues-assessing-adverse-impact-software-algorithms-and-artificial.pdf

[ref8] El Achmar D Bhagat R : The conceptual relation between human resource management (HRM) and competency mapping. *International Journal of Teaching & Education.* 2023.

[ref32] European Union Parliament and Council : *Regulation (EU) 2024/1689 … (Artificial Intelligence Act).*Official Journal of the European Union, OJ L, 2024/1689, 12.7.2024. EUR-Lex.2024. https://data.europa.eu/eli/reg/2024/1689/oj

[ref9] Fadhli M : Manajemen peningkatan mutu pendidikan. *Tadbir: Jurnal Studi Manajemen Pendidikan.* 2017;1(2):215–240. 10.29240/jsmp.v1i2.295

[ref10] Firza F Sarjono S : Penerapan algoritma K-Means dalam metode clustering untuk peminatan jurusan bagi siswa Swasta Pelita Raya Kota Jambi. *Jurnal Manajemen Sistem Informasi.* 2020;5(3):371–382.

[ref11] Gangl M : Labor market structure and re-employment rates: Unemployment dynamics in West Germany and the United States. *Research in Social Stratification and Mobility.* 2003;20:185–224. 10.1016/S0276-5624(03)20004-4

[ref12] Gie W Jollyta D : Perbandingan Euclidean dan Manhattan untuk optimasi cluster menggunakan Davies-Bouldin Index: Status COVID-19 wilayah Riau. *Prosiding Seminar Nasional Riset Information Science (SENARIS).* 2020, July;2:187–191.

[ref13] Green AE Hoyos M Li Y : *Job search study: Literature review and analysis of the Labour Force Survey.* London: Department for Work and Pensions;2011.

[ref14] Hurbean L Miliaru F Muntean M : The impact of business intelligence and analytics adoption on decision-making effectiveness and managerial work performance. *Scientific Annals of Economics and Business.* 2023;70:43–54. 10.47743/saeb-2023-0012

[ref15] Irmawan I Sagharmata FA Ruthriana F : Analisis dampak pembangunan Kota Hutan (Forest City) (Studi kasus: Ibu Kota Nusantara (IKN), Kalimantan). *Prosiding Seminar Rekayasa Teknologi (SemResTek).* 2023;299–304.

[ref16] Jain AK Murty MN Flynn PJ : Data clustering: A review. *ACM Computing Surveys (CSUR).* 1999;31(3):264–323. 10.1145/331499.331504

[ref17] Jaya DJ : Supplementary Materials R1 for “Application of K-Means Clustering for Job Applicant Analysis in Construction Firms Using R”.[Data set]. *Zenodo.* 2026. 10.5281/zenodo.18501546 PMC13294664 42368335

[ref18] Kassambara A : *Practical guide to cluster analysis in R.* STHDA; 1st ed. 2017. Reference Source

[ref19] London HH : *Principles and techniques of vocational guidance.* Ohio: Charles E. Merrill Publishing Company;1973.

[ref20] Manikandan S Caroline AL Kanniamma D : The study on clustering analysis in data mining. *International Journal of Data Mining Techniques and Applications.* 2018;7(1):46–49.

[ref33] National Institute of Standards and Technology (NIST) : *Artificial Intelligence Risk Management Framework (AI RMF 1.0).* 2023. 10.6028/NIST.AI.100-1

[ref21] Pala SK : Use and applications of data analytics in human resource management and talent acquisition. *International Journal of Enhanced Research in Science, Technology & Engineering.* 2021;10:2319–7463.

[ref22] Purba W Tamba S Saragih J : The effect of mining data K-Means clustering toward students profile model drop out potential. *IOP Conference Series: Journal of Physics.* 2018;1007(1):012046–012049. 10.1088/1742-6596/1007/1/012049

[ref23] Rawat A Nadavulakere S Isenhour L : Career enhancement strategies, supportive work relationships and subjective career success: The moderating role of family–work conflict. *Career Dev. Int.* 2024;29(4):421–433. 10.1108/CDI-06-2023-0160

[ref24] Smith SC Todaro MP : *Economic development.* Boston: Pearson Education; 12th ed. 2015.

[ref25] Supriyanti SS Kusmayanti JD Paluseri ARA : Pemberdayaan masyarakat sekitar di wilayah Ibu Kota Nusantara. *Masyarakat Indonesia.* 2023;49(1):93–102.

[ref26] Van der Heijden BIJM Hofer A Semeijn J : “Don’t you worry ’bout a thing” – The moderating role of age in the relationship between qualitative job insecurity and career sustainability. *Career Dev. Int.* 2024;29(5):527–543. 10.1108/CDI-08-2023-0280

[ref27] Widiyaningtyas T Prabowo MIW Pratama MAM : Implementation of K-Means clustering to distribution of high school teachers. *Proceeding EECSI, Yogyakarta, 19–21 September.* 2017, September;49–54.

[ref28] Widodo SE : *Manajemen pengembangan sumber daya manusia.* Yogyakarta: Pustaka Pelajar;2018.

[ref29] Wiharto W Suryani E : The comparison of clustering algorithms K-Means and Fuzzy C-Means for segmentation retinal blood vessels. *Acta Informatica Medica.* 2020;28(1):42–46. 10.5455/aim.2020.28.42-47 32210514 PMC7085333

[ref30] Zhang M Zhou S Wu Y : Pressure from social media: Influence of social media usage on career exploration. *Career Dev. Int.* 2024;29(1):93–112. 10.1108/CDI-01-2023-0016

