# Application of K-Means Clustering for Job Applicant Analysis in Construction Firms Using R

**DOI:** 10.12688/f1000research.172383.3

**Published:** 2026-06-16

**Authors:** Daniel Jesayanto Jaya, Wahyu Muhammad Ramdhani, Endang Wati, Yogi Novario Nandes, Ilma Zahriyatun Nadhiroh, Reza Bakhrun Fidianto Pade

**Affiliations:** 1 Technology and Vocational Education and Training, Universitas Negeri Yogyakarta, Yogyakarta, Special Region of Yogyakarta, 55282, Indonesia; 2 Building Engineering Education, Universitas Negeri Jakarta, East Jakarta, Special Capital Region of Jakarta, Indonesia; 3 Educational Research and Evaluation, Universitas Negeri Yogyakarta, Yogyakarta, Special Region of Yogyakarta, 55282, Indonesia; 4 English Language Education, Universitas Negeri Yogyakarta, Yogyakarta, Special Region of Yogyakarta, 55282, Indonesia

**Keywords:** K-Means Clustering; data-driven recruitment; workforce selection; cluster visualization; construction competencies

## Abstract

This study applies K-Means clustering to segment job applicant test data from a construction consulting firm to support data-driven screening decisions. From 161 applicants, 30 candidates who met the document-screening requirements were invited for in-person testing and included in the analysis. Three assessment variables were used: AutoCAD drafting skills, planning/supervision report-writing skills, and adaptability. Using R, K-Means clustering was performed to partition candidates into three groups based on multivariate similarity patterns, and the resulting group structure was visualized using 2D and 3D scatter plots. The clustering output revealed distinct competency profiles: one group characterized by generally lower scores across the three variables, a second group with moderate and mixed scores, and a third group with consistently higher scores. Internal validity indices suggested modest separation (mean silhouette = 0.16; Davies–Bouldin Index = 2.05), consistent with exploratory clustering on a small pre-screened sample. These patterns provide a structured interpretation of applicant diversity and can inform practical recruitment actions such as prioritizing candidates for interviews, identifying borderline profiles for additional evaluation, and designing targeted upskilling recommendations for specific competency gaps. Overall, this study illustrates how unsupervised clustering of routine recruitment test results may support more structured interpretation of applicant competency profiles in early-stage construction-sector recruitment, provided that the results are used cautiously alongside professional judgment and further validation.

## 1. Introduction

### 1.1 Research background

In the modern workplace, workforce selection is a critical component of human resource development, particularly in sectors that require a combination of technical expertise and adaptive capability. Career development and career transformation are influenced not only by formal qualifications but also by individuals’ ability to adapt to changing work environments and collaborate effectively with diverse stakeholders. Data-driven approaches to workforce analysis have therefore gained attention as tools to support more structured and transparent evaluation processes ( Pala, 2021).

Recruitment involves more than sourcing candidates; it requires systematic decision-making informed by job analysis, organizational needs, and available labor characteristics ( Widodo, 2018). Job analysis plays a central role in defining task requirements, competency expectations, and qualification standards, thereby helping organizations align applicants with role-specific demands. From the applicant’s perspective, successful job search outcomes depend on understanding personal competencies, evaluating labor market opportunities, and developing skills that match employer expectations ( London, 1973).

In the construction sector, technical competencies such as AutoCAD drafting, the ability to prepare planning and supervision reports, and adaptability to dynamic project environments are particularly valued ( Gangl, 2003). These competencies are increasingly important in large-scale infrastructure development contexts. In Indonesia, national strategic projects such as the Nusantara Capital City (Ibu Kota Nusantara, IKN) development have intensified demand for construction personnel with both technical proficiency and social adaptability ( Irmawan et al., 2023; Supriyanti et al., 2023). Managing and interpreting recruitment assessment data in such contexts presents practical challenges, especially when organizations must evaluate multiple competency dimensions simultaneously.

Contemporary recruitment is increasingly shaped by data-driven and technology-assisted decision-making. In human resource management, analytics can help organizations organize multidimensional applicant information, improve the transparency of assessment processes, and support more systematic screening decisions ( Pala, 2021; Hurbean et al., 2023; Madanchian, 2024). However, the use of analytical tools in recruitment also requires caution because applicant evaluation is a consequential decision-making context. Recent discussions on AI-assisted hiring and algorithmic decision-making emphasize that analytics should not be treated as a substitute for professional judgment, particularly when sample size, assessment scope, and validation evidence are limited ( Rigotti & Fosch-Villaronga, 2024; Dadaboyev et al., 2025). Issues such as transparency, explainability, fairness, adverse impact, and human oversight are central to responsible recruitment analytics ( National Institute of Standards and Technology [NIST], 2023; European Union Parliament and Council, 2024; U.S. Equal Employment Opportunity Commission [EEOC], 2023).

In this study, K-Means clustering is therefore positioned as an exploratory decision-support technique rather than an automated hiring or rejection system. The purpose of clustering is to describe competency patterns among candidates who had already passed document screening and completed in-person assessment. The resulting cluster labels are analytical interpretations of score similarity patterns and should be read as preliminary competency profiles that may inform further managerial review, not as definitive employment decisions ( Jain et al., 1999; Kassambara, 2017).

Cluster analysis offers a data-driven approach to explore patterns within applicant assessment data by grouping individuals with similar characteristics. Clustering techniques partition data into internally homogeneous and externally heterogeneous groups, thereby supporting structured interpretation of complex multivariate information ( Jain et al., 1999). Among these techniques, K-Means clustering is widely used due to its computational simplicity and interpretability, making it suitable for exploratory analysis of recruitment-related datasets. In recruitment contexts, clustering can be applied to post-screening assessment data to identify competency profiles rather than to make automated hiring decisions.

Beyond operational efficiency, the use of data-driven tools in recruitment raises broader issues of transparency, governance, and fairness in algorithm-assisted selection. International guidance emphasizes that AI-enabled assessment should be accompanied by risk management, documentation, and ongoing monitoring of unintended impacts ( NIST, 2023). In addition, U.S. Equal Employment Opportunity Commission (EEOC) guidance highlights that employers should assess whether algorithmic or AI-based selection procedures produce adverse impact under Title VII and aligns such assessment with the Uniform Guidelines on Employee Selection Procedures ( EEOC, 2023). Similarly, the European Union Artificial Intelligence Act classifies certain AI systems used in employment-related contexts as high-risk, reinforcing expectations for accountability and safeguards when analytics influence employment decisions ( European Union Parliament and Council, 2024). Accordingly, this study positions K-Means clustering as an exploratory decision-support technique rather than an automated hiring system; cluster labels are interpreted cautiously as descriptive competency profiles and are intended to complement human review rather than replace managerial judgment.

This study applies K-Means clustering to recruitment test data from a construction consulting firm, focusing on candidates who passed document screening and completed in-person assessments. Using three core variables—AutoCAD drafting skills, planning/supervision report-writing skills, and adaptability—the study demonstrates how unsupervised clustering can support exploratory analysis of applicant competency profiles within a real organizational context.

### 1.2 Literature review

Clustering is an unsupervised analytical technique used to group objects into clusters based on attribute similarity, such that objects within the same cluster exhibit higher similarity than those in other clusters ( Jain et al., 1999). By minimizing within-cluster variation and maximizing between-cluster differences, clustering supports pattern discovery and interpretation in complex datasets ( Manikandan et al., 2018; Darmi & Setiawan, 2016). For organizational and workforce analytics, clustering provides a data-driven means of understanding heterogeneity among individuals without requiring predefined class labels.

Among various clustering approaches, K-Means clustering is one of the most widely applied methods due to its simplicity, efficiency, and interpretability. K-Means partitions data into *k* clusters by iteratively assigning observations to the nearest centroid and updating centroid positions until convergence is achieved ( Jain et al., 1999). Because of its relatively low computational cost, K-Means is suitable for applied settings where rapid analysis and transparent interpretation are required ( Fadhli, 2017).

Previous studies demonstrate applicability across domains. In educational research, K-Means has been used to analyze student preferences and learning achievement patterns ( Firza & Sarjono, 2020). In organizational contexts, it has been applied to group employees based on discipline and performance indicators to support human resource decision-making ( Agustina & Prihandoko, 2018). Comparative studies suggest that while alternatives such as Fuzzy C-Means may offer advantages in some conditions, K-Means remains computationally efficient and practical for many real-world applications ( Wiharto & Suryani, 2020).

**1.2.1 K-Means algorithm**

K-Means is a partition-based clustering algorithm that divides data into a predefined number of clusters by minimizing the average distance between data points and their respective cluster centroids ( Widiyaningtyas et al., 2017). The algorithm operates iteratively, beginning with the selection of initial centroid values and proceeding through repeated reassignment of data points based on distance calculations until cluster membership stabilizes ( Purba et al., 2018). Prior work emphasizes that K-Means can be sensitive to initialization and the scale of input variables, highlighting the need for transparent methodological choices in applied studies ( Jain et al., 1999).

**1.2.2 Worker recruitment**

Recruitment is a strategic organizational process aimed at attracting and selecting individuals whose competencies align with job requirements and organizational objectives. Job analysis plays a critical role in defining tasks, responsibilities, and qualification standards, thereby guiding recruitment and selection decisions ( Widodo, 2018). In the construction sector, recruitment emphasizes a combination of technical competencies—such as drafting and report preparation—and adaptive capabilities, reflecting the dynamic and collaborative nature of construction projects ( Gangl, 2003). The job search process seeks to match job seekers with appropriate opportunities and can be supported through technology-enabled and data-driven methods ( Green et al., 2011). Given the multidimensionality of applicant data, clustering methods such as K-Means offer a way to organize assessment results into interpretable competency profiles that can support early-stage evaluation ( Jain et al., 1999).

**1.2.3 HR analytics and algorithm-assisted recruitment**

HR analytics refers to the systematic use of workforce-related data to support organizational decision-making. In recruitment, HR analytics can assist decision-makers by structuring applicant information, identifying patterns across competency dimensions, and supporting more consistent interpretation of assessment results ( Pala, 2021; Hurbean et al., 2023; Venugopal et al., 2024). This is particularly relevant in sectors such as construction consulting, where applicants may need to demonstrate both technical skills and adaptive capabilities. From a human resource development perspective, recruitment is not only a selection activity but also part of a broader workforce capability system because it determines how organizations identify, develop, and allocate human talent ( Widodo, 2018; El Achmar & Bhagat, 2023; Jaya et al., 2026a).

The growing use of analytics and artificial intelligence in recruitment has also generated debate about fairness, transparency, and accountability. AI-assisted hiring systems may improve efficiency in screening and assessment, but recent literature cautions that these systems can reproduce bias, create opacity in decision-making, and produce adverse impacts when used without proper validation and oversight ( Madanchian, 2024; Rigotti & Fosch-Villaronga, 2024; Dadaboyev et al., 2025). Therefore, algorithm-assisted recruitment should be accompanied by clear documentation, human review, and careful interpretation of results ( NIST, 2023; EEOC, 2023; European Union Parliament and Council, 2024).

In this context, clustering offers a comparatively transparent exploratory method. Unlike predictive models that estimate hiring outcomes or job performance, clustering groups applicants based on similarity in observed assessment scores ( Jain et al., 1999; Kassambara, 2017). This makes the method useful for descriptive segmentation and early-stage decision support. Nevertheless, cluster labels should not be interpreted as evidence of actual job performance or recruitment effectiveness unless they are externally validated using hiring outcomes, supervisor evaluations, or post-employment performance data ( Jaya et al., 2026b). Accordingly, this study uses K-Means clustering to generate interpretable applicant competency profiles while acknowledging the methodological and ethical limits of using analytical tools in recruitment decision-making.

## 2. Methods

### 2.1 Research design

**Figure 1.**
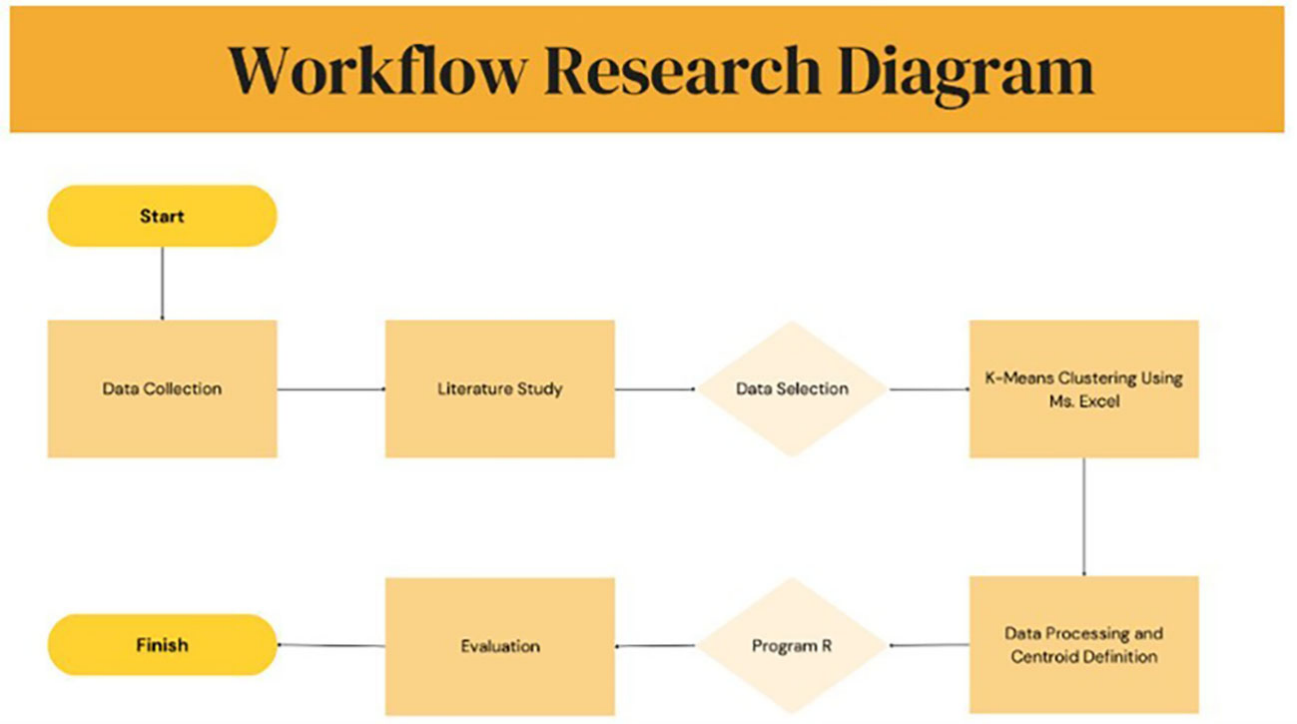
Workflow research diagram.

This study employed a quantitative, exploratory research design using unsupervised clustering to analyze recruitment assessment data from a construction consulting firm. The primary objective was to explore competency-based grouping patterns among job applicants using K-Means clustering as a decision-support tool, rather than to predict hiring outcomes or evaluate post-employment performance. The overall research workflow is illustrated in Figure 1.

### 2.2 Data source and participant selection

The data were obtained from CV Ardantama Putra Perkasa as part of its internal recruitment process. Although the vacancy was advertised through JobStreet Indonesia, all data analyzed in this study originated exclusively from the company’s internal screening and testing procedures.

A total of 161 applicants applied for the position. Applicants were shortlisted through the company’s standard document-screening procedure conducted by the HR team and the hiring unit. Screening focused on administrative completeness and role relevance, including: (i) completeness of required documents; (ii) educational background and relevance to construction consulting work; (iii) evidence of relevant technical exposure, such as drafting/reporting-related tasks or portfolio where available; and (iv) basic eligibility criteria specified in the vacancy announcement. From this process, 30 candidates met the minimum document-screening requirements and were invited to complete in-person competency testing.

Only these 30 shortlisted candidates were included in the clustering analysis because complete assessment scores were available for all three variables: AutoCAD drafting skills, planning/supervision report-writing skills, and adaptability. This sampling decision means that the analysis represents competency patterns among a pre-screened analytical sample rather than the full applicant pool. Following STROBE-style reporting principles, this sampling boundary is explicitly stated to clarify the analytical population, eligibility process, and limitations of inference ( von Elm et al., 2007). The results therefore should not be generalized to all 161 applicants or to construction job applicants more broadly. Instead, the analysis illustrates how clustering can be used to organize assessment data after an initial administrative screening stage has already occurred.

### 2.3 Assessment variables

Candidates were evaluated using three competency indicators relevant to construction consulting roles:

1. AutoCAD drafting skills;
2. planning/supervision report-writing skills;
3. adaptability.

AutoCAD drafting skills refer to candidates’ ability to produce and interpret technical drawings using AutoCAD. Planning/supervision report-writing skills refer to candidates’ ability to prepare structured reports related to construction planning and supervision activities. Adaptability refers to candidates’ ability to adjust to changing project conditions, work demands, and organizational expectations. These indicators reflect the need to combine technical competence with adaptive and work-relevant capability in construction-related occupational settings ( Gangl, 2003; Widodo, 2018; Jaya et al., 2026a).

Each variable was assessed on a numerical scale from 0 to 100, with higher scores indicating stronger performance. Because all three variables used the same scale, the raw scores were retained for clustering analysis to preserve the original meaning of the assessment results.

### 2.4 Data preprocessing, outlier, and sensitivity checks

The dataset was reviewed for completeness and consistency before clustering. All 30 shortlisted candidates had complete scores across the three assessment variables; therefore, no candidate records were removed because of missing data. The variable names were standardized throughout the dataset and manuscript as AutoCAD drafting skills, planning/supervision report-writing skills, and adaptability. Clear reporting of data eligibility, exclusions, and analytical decisions is important for reproducibility in observational data analysis ( von Elm et al., 2007).

Because all variables were measured on the same 0–100 scale, the analysis used raw scores without additional normalization or standardization. This decision was made to preserve the practical interpretation of the original assessment scores. Euclidean distance was used as the distance metric because K-Means clustering groups observations by minimizing within-cluster squared distances ( Jain et al., 1999; Kassambara, 2017). Basic outlier and sensitivity checks were conducted by inspecting score distributions, distances to cluster centroids, and two-dimensional and three-dimensional visualizations. These checks were used to determine whether any individual observation disproportionately shaped the cluster interpretation.

### 2.5 Clustering procedure

K-Means clustering was applied to group candidates based on similarity across the three assessment variables. The number of clusters was set to k = 3 because the company required an interpretable decision-support structure that could distinguish lower, intermediate, and higher competency profiles for managerial review. However, this three-cluster solution is not interpreted as proof that the dataset contains three naturally distinct applicant groups. Rather, k = 3 was used as a practically meaningful segmentation structure and was examined using internal diagnostic checks.

The analysis used Euclidean squared distance to assign each candidate to the nearest centroid. Because K-Means can be sensitive to centroid initialization, variable scaling, and the predefined number of clusters, the initial centroids and iteration procedure were explicitly reported in the supplementary materials. Methodological transparency is particularly important when applying K-Means to small applied datasets ( Jain et al., 1999; Kassambara, 2017). Cluster-number justification was examined using the elbow method and additional internal diagnostics, including the silhouette coefficient and Davies–Bouldin Index ( Rousseeuw, 1987; Davies & Bouldin, 1979). These diagnostics were used to assess whether the three-cluster solution was reasonably interpretable while acknowledging that internal validation metrics in small, pre-screened datasets should be interpreted cautiously.

The clustering workflow was implemented using spreadsheet-based calculations for transparency of manual steps and R programming for reproducibility, validity checks, and visualization. Intermediate iteration tables, R scripts, and visualization outputs are provided as extended data.

The final clustering procedure was implemented in R using a custom K-Means function with fixed initial centroids derived from the spreadsheet-based clustering workflow. The algorithm calculated Euclidean squared distances, assigned each candidate to the nearest centroid, recalculated cluster centroids, and repeated the process until centroid values stabilized. Because the initial centroids were fixed and explicitly reported, the clustering procedure is deterministic for the reported dataset. The R script, supporting tables, and visualization outputs are provided as extended data.

### 2.5.1 Cluster-number justification and stability checks

Because the number of clusters in K-Means must be specified before analysis, the choice of k was evaluated using both practical and diagnostic considerations. Practically, k = 3 corresponded to the company’s need for three interpretable competency profiles that could support recruitment discussion: lower, intermediate, and higher competency profiles. Analytically, the three-cluster solution was examined using the elbow method, silhouette coefficient, and Davies–Bouldin Index.

The elbow method was used to compare the reduction in within-cluster sum of squares across alternative cluster numbers. The silhouette coefficient was used to evaluate the degree to which candidates were closer to their assigned cluster than to other clusters ( Rousseeuw, 1987). The Davies–Bouldin Index was used to assess within-cluster compactness relative to between-cluster separation ( Davies & Bouldin, 1979). Because the final workflow used fixed initial centroids, the analysis was reproducible without stochastic initialization. The diagnostic indices were therefore used primarily to evaluate the interpretability of the selected three-cluster solution rather than to claim strong natural cluster separation. These diagnostic checks are commonly recommended because internal validity indices provide useful but incomplete evidence, particularly when clusters overlap or sample sizes are small ( Jain et al., 1999; Kassambara, 2017; Jaya et al., 2026b).

These diagnostics were not used to claim that k = 3 represents a definitive natural structure in the data. Instead, they were used to examine whether the selected three-cluster solution was defensible as an exploratory and managerially interpretable grouping of shortlisted candidates.

### 2.6 Visualization and interpretation

Clustering results were visualized using two-dimensional and three-dimensional scatter plots. Two-dimensional plots illustrated relationships between AutoCAD drafting skills and planning/supervision report-writing skills, while three-dimensional plots incorporated adaptability as a third axis.

Clusters were subsequently labeled as “Lower competency profile,” “Intermediate/mixed competency profile,” and “Higher competency profile” based on their relative position in the multivariate competency space. These labels represent analytical interpretations of score patterns and do not constitute formal hiring decisions made by the company.

### 2.7 Scope and methodological limitations

This study focuses on exploratory grouping of recruitment assessment data from a pre-screened subset of applicants. The clustering results were not validated against final hiring decisions or post-employment performance outcomes. Accordingly, findings should be interpreted as structured analytical support rather than definitive evidence of selection effectiveness.

### 2.8 Cluster validity assessment

To provide quantitative support for the cluster structure, internal validity indices were calculated. The silhouette coefficient was computed using Euclidean distances to estimate how well each candidate matched its assigned cluster relative to other clusters. The Davies–Bouldin Index (DBI) was calculated to evaluate average cluster similarity based on within-cluster dispersion relative to between-cluster centroid distances. These indices were interpreted as descriptive diagnostics of separation quality rather than evidence of predictive utility.

## 3. Results and discussion

### 3.1 Applicant characteristics

This study analysed recruitment assessment records from CV Ardantama Putra Perkasa, obtained from the company’s internal testing and selection process. A total of 161 applicants submitted applications, of whom 30 candidates meeting minimum screening criteria were invited for in-person testing. Each candidate was assessed on three indicators measured on a 0–100 scale: AutoCAD drafting skills (X), planning/supervision report-writing skills (Y), and adaptability (Z). Candidate characteristics and scores are summarised in Table 1.

**Table 1.** Shortlisted candidate characteristics and assessment scores.

Respondent code	Gender	AutoCAD drafting skills (X)	Planning/supervision report-writing skills (Y)	Adaptability (Z)
Resp1	Female	92	75	68
Resp2	Male	68	65	66
Resp3	Male	73	86	87
Resp4	Male	69	74	73
Resp5	Male	78	72	91
Resp6	Female	84	90	92
Resp7	Male	69	76	87
Resp8	Female	95	73	76
Resp9	Female	90	80	85
Resp10	Male	68	82	68
Resp11	Male	63	75	71
Resp12	Male	75	93	77
Resp13	Female	62	72	68
Resp14	Male	90	61	72
Resp15	Female	84	63	90
Resp16	Female	94	70	89
Resp17	Female	73	87	80
Resp18	Female	71	73	95
Resp19	Female	93	62	70
Resp20	Male	90	68	89
Resp21	Female	87	94	87
Resp22	Male	60	90	64
Resp23	Female	65	64	93
Resp24	Male	69	84	75
Resp25	Male	66	63	72
Resp26	Male	95	85	93
Resp27	Male	75	80	83
Resp28	Male	92	85	93
Resp29	Male	71	71	85
Resp30	Male	92	61	88

Overall, the score distribution shows meaningful heterogeneity across candidates—particularly in adaptability and planning/supervision report-writing—indicating variation in both technical and interpersonal readiness. This variability provides a suitable basis for exploratory clustering analysis.

### 3.2 K-Means clustering results

Using K-Means clustering with k = 3, the 30 assessed candidates were grouped based on similarity across AutoCAD drafting skills, planning/supervision report-writing skills, and adaptability. The three-cluster solution was selected because it provided a practically interpretable structure for recruitment discussion while remaining consistent with the exploratory purpose of the study. The clusters should therefore be interpreted as descriptive competency profiles rather than as statistically definitive applicant classes or formal hiring decisions ( Jain et al., 1999; Kassambara, 2017).

The first cluster represents candidates with comparatively lower overall competency profiles across the assessed variables. The second cluster represents candidates with mixed or intermediate competency profiles, indicating that further assessment or managerial consideration may be appropriate. The third cluster represents candidates with comparatively stronger combined technical and adaptive competency profiles. These labels are interpretive and intended to support structured review rather than automate recruitment outcomes, consistent with responsible use of analytics in consequential employment-related decisions ( NIST, 2023; EEOC, 2023; European Union Parliament and Council, 2024).

The first cluster is characterized by relatively lower combined scores across the three assessed competencies. The second cluster consists of candidates with moderate and mixed competency scores, reflecting intermediate profiles that may warrant further evaluation. The third cluster comprises candidates with comparatively higher scores across technical and adaptive dimensions, indicating stronger and more balanced competency profiles.

The clustering process involved iterative centroid updates until cluster memberships stabilized. To maintain readability, detailed distance-to-centroid and iteration tables are provided as extended data, while the main text reports the final centroid summary and stabilized cluster assignment.

The final stabilized cluster assignment is presented in Table 3. Respondent codes correspond to the candidate codes reported in Table 1. Detailed distance-to-centroid calculations and iteration outputs are provided in the Zenodo supplementary materials to support reproducibility without overloading the main manuscript. The final cluster centroid and size summary for the three interpretive profiles is presented in Table 2.

**Table 2.** Cluster centroid and size summary.

Cluster	n	AutoCAD drafting skills, mean	Planning/supervision report-writing skills, mean	Adaptability, mean	Interpretive profile
1	8	65.13	73.13	71.88	Lower competency profile
2	11	79.45	75.73	80.00	Intermediate/mixed competency profile
3	11	87.09	77.82	88.36	Higher competency profile

**Table 3.** Final cluster assignment by respondent code.

Respondent code	Cluster	Category
Resp2	1	Lower competency profile
Resp4	1	Lower competency profile
Resp10	1	Lower competency profile
Resp11	1	Lower competency profile
Resp13	1	Lower competency profile
Resp22	1	Lower competency profile
Resp25	1	Lower competency profile
Resp23	1	Lower competency profile
Resp24	2	Intermediate/mixed competency profile
Resp17	2	Intermediate/mixed competency profile
Resp12	2	Intermediate/mixed competency profile
Resp29	2	Intermediate/mixed competency profile
Resp18	2	Intermediate/mixed competency profile
Resp3	2	Intermediate/mixed competency profile
Resp27	2	Intermediate/mixed competency profile
Resp14	2	Intermediate/mixed competency profile
Resp19	2	Intermediate/mixed competency profile
Resp1	2	Intermediate/mixed competency profile
Resp30	2	Intermediate/mixed competency profile
Resp7	3	Higher competency profile
Resp5	3	Higher competency profile
Resp15	3	Higher competency profile
Resp6	3	Higher competency profile
Resp8	3	Higher competency profile
Resp9	3	Higher competency profile
Resp16	3	Higher competency profile
Resp20	3	Higher competency profile
Resp21	3	Higher competency profile
Resp26	3	Higher competency profile
Resp28	3	Higher competency profile

### 3.2.1 Cluster validity metrics

Internal validity diagnostics were calculated to evaluate the interpretability of the selected cluster solution. For the three-cluster solution, the mean silhouette coefficient was 0.16, indicating modest and weak-to-moderate separation among applicant competency profiles. The Davies–Bouldin Index was 2.05, suggesting limited compactness and separation between clusters. These values indicate that the clusters are interpretable for exploratory and managerial discussion, but they do not demonstrate strong natural separation in the data.

The elbow method was also used to compare the reduction in within-cluster sum of squares across alternative cluster numbers. The elbow pattern did not provide decisive evidence that three clusters represented a clearly optimal natural structure. Therefore, the three-cluster solution was retained primarily because it aligned with the company’s need for a practical and interpretable decision-support structure, while the internal validity indices were interpreted cautiously. These indicators are useful for assessing internal cluster structure, although they do not provide external validation of hiring effectiveness or job performance outcomes ( Davies & Bouldin, 1979; Rousseeuw, 1987; Jain et al., 1999).

### 3.3 Visualization of cluster structure

**Figure 2.**
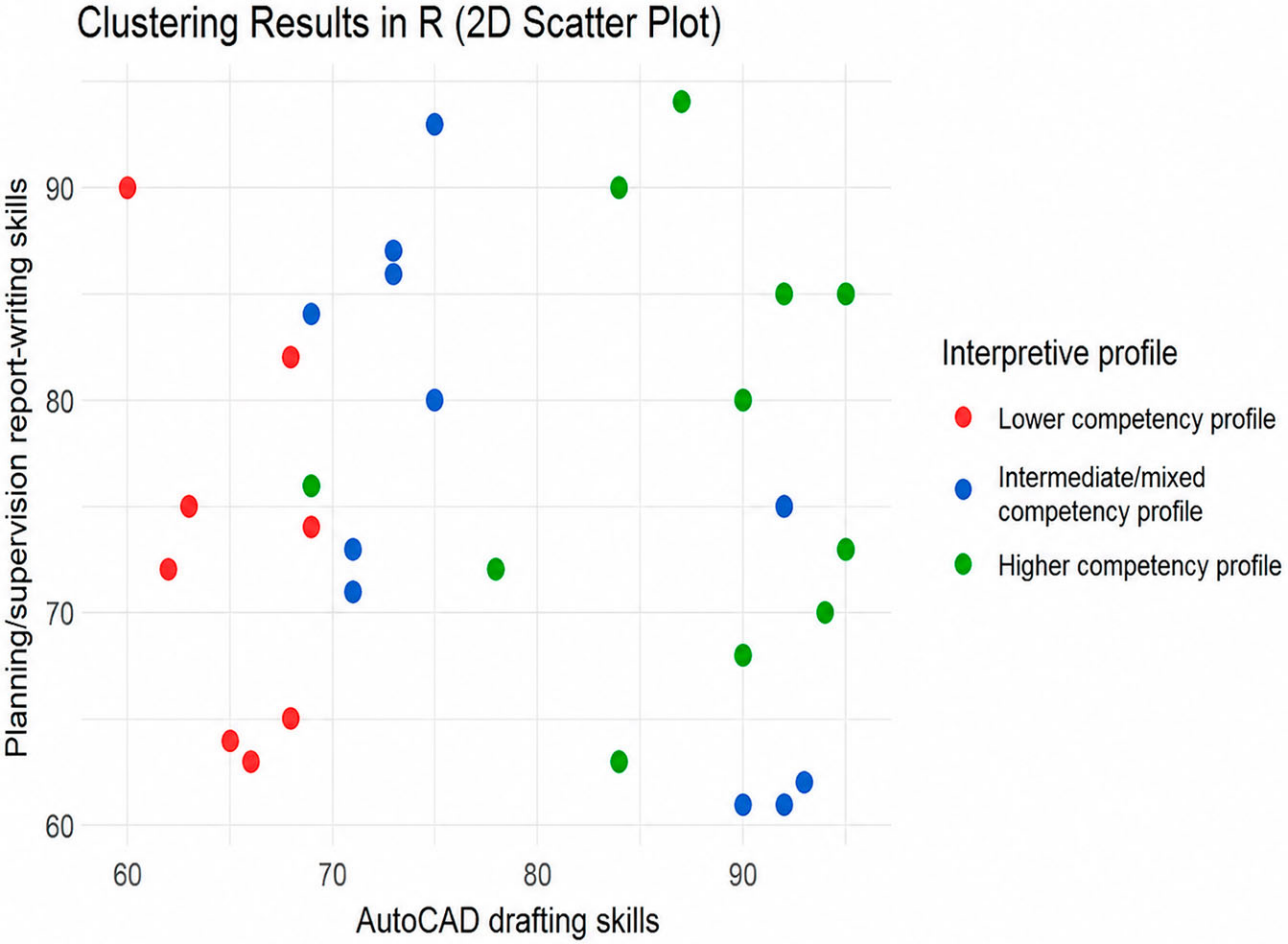
K-means clustering visualization in a 2D scatter plot.

To support interpretation, two-dimensional and three-dimensional visualizations were generated. Figure 2 presents a 2D scatter plot based on AutoCAD drafting skills and planning/supervision report-writing skills, showing visible separation between lower, intermediate, and higher competency profiles along key technical dimensions.

**Figure 3.**
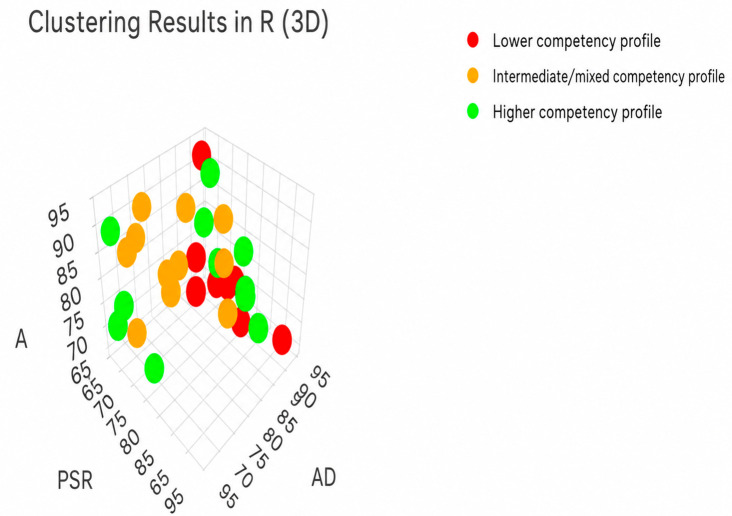
K-means clustering visualization in a 3D scatter plot.

Figure 3 extends the visualization into three dimensions by incorporating adaptability as a third axis. The 3D scatter plot reveals clearer spatial separation among clusters, particularly distinguishing candidates who combine strong technical skills with high adaptability from those with lower overall competency scores.

**Figure 4.**
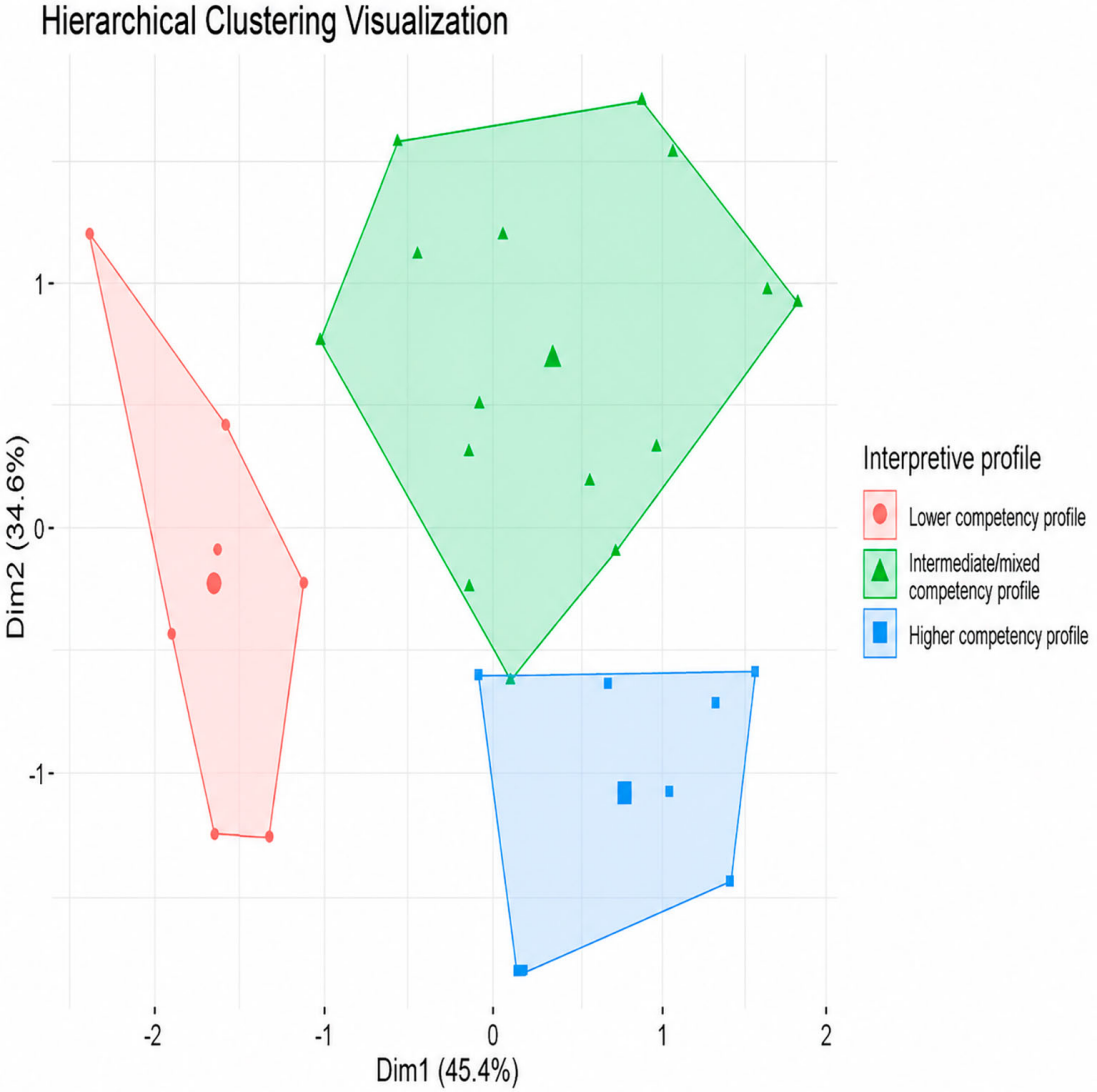
Hierarchical clustering visualization using PCA-projected dimensions.

To further examine structural consistency, hierarchical clustering projected onto principal component space is presented in Figure 4. Although hierarchical clustering was not employed as the primary analytical method, the observed grouping patterns broadly align with the K-Means classification, providing additional support for the stability of the three-cluster structure within this dataset.

### 3.4 Interpretation and discussion

The clustering results illustrate how K-Means can be used as an exploratory tool to organize recruitment assessment data into interpretable competency profiles within a construction consulting context. The findings suggest that candidates can be descriptively grouped according to combinations of technical and adaptive competencies. However, the results should not be interpreted as evidence that clustering improves recruitment effectiveness because the analysis was conducted on a small, pre-screened sample and was not externally validated against final hiring decisions, supervisor evaluations, or post-employment performance outcomes. This interpretation is consistent with methodological cautions in clustering research, where internal cluster structure does not automatically demonstrate practical or predictive validity ( Jain et al., 1999; Kassambara, 2017; Jaya et al., 2026b).

The intermediate cluster is particularly important from a managerial perspective because it represents candidates with mixed competency profiles. Rather than treating this group as a fixed decision category, organizations may use it to identify applicants who require follow-up interviews, additional assessment, or targeted development consideration. In this sense, clustering functions as a decision-support lens that helps structure discussion but does not replace professional judgment ( Pala, 2021; Hurbean et al., 2023; Madanchian, 2024).

From an ethical and governance perspective, the use of analytics in recruitment should remain transparent, documented, and subject to human oversight. Cluster labels such as “Lower competency profile,” “Intermediate/mixed competency profile,” and “Higher competency profile” should be understood as descriptive analytical labels rather than automated employment decisions. When analytical tools are used in recruitment contexts, organizations should ensure that their use is aligned with fairness, accountability, and validation principles ( Rigotti & Fosch-Villaronga, 2024; NIST, 2023; EEOC, 2023; European Union Parliament and Council, 2024).

### 3.5 Methodological considerations and limitations

Several limitations should be considered when interpreting these findings. First, the analysis was limited to 30 candidates who had already passed document screening and completed in-person testing. Therefore, the results describe competency patterns within a pre-screened analytical sample and cannot be generalized to the full pool of 161 applicants. Second, the sample size was small for clustering analysis, which limits the strength of claims regarding cluster stability and natural group structure. Third, the study relied on three assessment variables only; additional indicators such as interview performance, portfolio quality, work experience, certification, or supervisor-rated performance could produce a more comprehensive applicant profile. These reporting boundaries are important for transparency in observational data analysis ( von Elm et al., 2007).

Fourth, the cluster results were not externally validated against actual hiring outcomes or post-employment job performance. As a result, the study cannot claim that the clustering procedure improves recruitment effectiveness. Instead, the findings should be interpreted as an illustrative application of clustering for organizing multidimensional assessment data. Future research should test this approach using larger applicant pools, additional competency indicators, longitudinal job-performance data, and fairness or adverse-impact analysis ( Jain et al., 1999; EEOC, 2023; Jaya et al., 2026b).

## 4. Conclusions

This study explored the use of K-Means clustering as an exploratory analytical approach for organizing recruitment assessment data in a construction consulting context. Using data from 30 shortlisted candidates who completed in-person testing, the analysis grouped applicants based on three competency indicators: AutoCAD drafting skills, planning/supervision report-writing skills, and adaptability.

The three-cluster solution provided an interpretable structure for describing lower, intermediate, and higher competency profiles among the shortlisted candidates. However, these clusters should not be interpreted as definitive hiring categories or as evidence of improved recruitment effectiveness. The analysis was based on a small, pre-screened sample and was not externally validated using final hiring decisions or post-employment performance outcomes.

The main contribution of this study is therefore methodological and illustrative. It shows how clustering can help structure multidimensional recruitment assessment data and support transparent discussion among decision-makers. Used appropriately, clustering may complement professional judgment by making applicant competency patterns easier to interpret. Nevertheless, the method should be applied cautiously, with clear documentation, human oversight, and further validation before being used in consequential recruitment decisions ( NIST, 2023; EEOC, 2023; European Union Parliament and Council, 2024).

Future studies should apply this approach to larger and more diverse applicant datasets, include additional competency and background variables, compare alternative clustering methods, and examine whether cluster membership relates to actual hiring outcomes or subsequent job performance. Further work should also consider fairness, transparency, and adverse-impact assessment when analytics are used to support recruitment decisions ( Rigotti & Fosch-Villaronga, 2024; Dadaboyev et al., 2025).

## Ethical approval

Ethical review and approval were not required for this study because the researchers analyzed fully anonymized secondary data that had been lawfully transferred by CV Ardantama Putra Perkasa under a formal Data Usage Agreement (No. 12/X/S-K/APP/2024). According to Indonesian national research ethics regulations (Permenkes RI No. 74/2016, Article 11) and the general principles of the Declaration of Helsinki, research involving secondary anonymized non-clinical data that cannot identify individuals is exempt from institutional ethical review. Therefore, this study qualifies for an ethics exemption.

## Informed consent

Informed consent for data use was not obtained directly by the researchers, as all data were collected by CV Ardantama Putra Perkasa under standard recruitment procedures. The company confirmed, through the Data Usage Agreement (No. 12/X/S-K/APP/2024), that job applicants had authorized the use of their anonymized recruitment test results for evaluation and administrative purposes in accordance with Indonesian data protection regulations (UU ITE and PP 71/2019). Because the researchers received only anonymized secondary data and had no access to identifiable information, this study meets the criteria for consent exemption.

## Data Availability

The anonymized job applicant dataset is not publicly available due to confidentiality agreements with CV Ardantama Putra Perkasa. Access may be granted for legitimate academic research upon reasonable request to the corresponding author ( danieljesayanto.2023@student.uny.ac.id), subject to approval by the data owner and compliance with Indonesian data protection regulations (UU ITE and PP 71/2019), including signing a Data Use Agreement and a commitment not to attempt re-identification. Extended data supporting this study, including R scripts, clustering iteration tables, visualizations, and documentation, are openly available in Zenodo at https://doi.org/10.5281/zenodo.20070567 ( Jaya, 2026) under the Creative Commons Attribution 4.0 International (CC BY 4.0) license.

## References

[ref1] Akkermans J Donald WE Jackson D : Are we talking about the same thing? The case for stronger connections between graduate and worker employability research. *Career Dev. Int.* 2024;29(1):80–92. 10.1108/CDI-08-2023-0278

[ref2] Agustina N Prihandoko P : Perbandingan algoritma K-Means dengan Fuzzy C-Means untuk clustering tingkat kedisiplinan kinerja karyawan. *Jurnal RESTI (Rekayasa Sistem dan Teknologi Informasi).* 2018;2(3):621–626. 10.29207/resti.v2i3.492

[ref3] Brown P Hesketh A : The mismanagement of talent: Employability and jobs in the knowledge economy. *Ind. Labor Relat. Rev.* 2005. 10.2189/asqu.2005.50.2.306

[ref4] Chen Yu : *K-Means clustering.* Indiana University;2020.

[ref5] Dadaboyev SMU Soomro TR Bakhsh FA : Role of artificial intelligence in employee recruitment: a systematic literature review. *Int. J. Inf. Technol.* 2025. 10.1007/s44282-025-00246-w

[ref6] Darmi Y Setiawan A : Penerapan metode clustering K-Means dalam pengelompokan penjualan produk. *Jurnal Media Infotama.* 2016;12(2):148–157.

[ref7] Davies DL Bouldin DW : A cluster separation measure. *IEEE Transactions on Pattern Analysis and Machine Intelligence.* 1979;PAMI-1:224–227. 10.1109/TPAMI.1979.4766909 21868852

[ref8] Diván M : Data-driven decision making. *2017 IEEE International Conference on Technological Innovations in ICT for Agriculture and Rural Development (TIAR).* IEEE;2017; pp.50–56. 10.1109/ICTUS.2017.8285973

[ref9] Donald WE Van der Heijden BIJM Manville G : (Re) Framing sustainable careers: Toward a conceptual model and future research agenda. *Career Dev. Int.* 2024;29(5):513–526. 10.1108/CDI-02-2024-0073

[ref10] EEOC : *Select Issues: Assessing Adverse Impact in Software, Algorithms, and Artificial Intelligence Used in Employment Selection Procedures Under Title VII of the Civil Rights Act of 1964 (Technical Assistance; **EEOC-NVTA-2023-2**, Issue Date: **2023-05-18**).* U.S. Equal Employment Opportunity Commission;2023. https://data.aclum.org/storage/2025/01/EOCC_www_eeoc_gov_laws_guidance_select-issues-assessing-adverse-impact-software-algorithms-and-artificial.pdf

[ref11] El Achmar D Bhagat R : The conceptual relation between human resource management (HRM) and competency mapping. *International Journal of Teaching & Education.* 2023.

[ref12] European Union Parliament and Council : *Regulation (EU) 2024/1689 … (Artificial Intelligence Act).*Official Journal of the European Union, OJ L, 2024/1689, 12.7.2024. EUR-Lex.2024. https://data.europa.eu/eli/reg/2024/1689/oj

[ref13] Fadhli M : Manajemen peningkatan mutu pendidikan. *Tadbir: Jurnal Studi Manajemen Pendidikan.* 2017;1(2):215–240. 10.29240/jsmp.v1i2.295

[ref14] Firza F Sarjono S : Penerapan algoritma K-Means dalam metode clustering untuk peminatan jurusan bagi siswa Swasta Pelita Raya Kota Jambi. *Jurnal Manajemen Sistem Informasi.* 2020;5(3):371–382.

[ref15] Gangl M : Labor market structure and re-employment rates: Unemployment dynamics in West Germany and the United States. *Research in Social Stratification and Mobility.* 2003;20:185–224. 10.1016/S0276-5624(03)20004-4

[ref16] Gie W Jollyta D : Perbandingan Euclidean dan Manhattan untuk optimasi cluster menggunakan Davies-Bouldin Index: Status COVID-19 wilayah Riau. *Prosiding Seminar Nasional Riset Information Science (SENARIS).* 2020, July;2:187–191.

[ref17] Green AE Hoyos M Li Y : *Job search study: Literature review and analysis of the Labour Force Survey.* London: Department for Work and Pensions;2011.

[ref18] Hurbean L Miliaru F Muntean M : The impact of business intelligence and analytics adoption on decision-making effectiveness and managerial work performance. *Scientific Annals of Economics and Business.* 2023;70:43–54. 10.47743/saeb-2023-0012

[ref19] Irmawan I Sagharmata FA Ruthriana F : Analisis dampak pembangunan Kota Hutan (Forest City) (Studi kasus: Ibu Kota Nusantara (IKN), Kalimantan). *Prosiding Seminar Rekayasa Teknologi (SemResTek).* 2023;299–304.

[ref20] Jain AK Murty MN Flynn PJ : Data clustering: A review. *ACM Computing Surveys (CSUR).* 1999;31(3):264–323. 10.1145/331499.331504

[ref21] Jaya DJ Sudira P Raharjo NE : Vocational teacher professionalisation as human resource development: policy reflections from Indonesia. *Int. J. Train. Res.* 2026a:1–11. 10.1080/14480220.2026.2630821

[ref22] Jaya DJ Ramdhani WM Hamid HWR : Integrating fuzzy C-means clustering and Random Forest for multivariate performance prediction in vocational education. *Commun. Stat. Case Stud. Data Anal. Appl.* 2026b. 10.1080/23737484.2026.2640437

[ref23] Jaya DJ : Supplementary Materials R2 for “Application of K-Means Clustering for Job Applicant Analysis in Construction Firms Using R”. [Data set]. *Zenodo.* 2026. 10.5281/zenodo.20070567 PMC13294664 42368335

[ref24] Kassambara A : *Practical guide to cluster analysis in R.* STHDA; 1st ed. 2017. Reference Source

[ref25] London HH : *Principles and techniques of vocational guidance.* Ohio: Charles E. Merrill Publishing Company;1973.

[ref26] Madanchian M : From Recruitment to Retention: AI Tools for Human Resource Decision-Making. *Appl Sci.* 2024;14(24):11750. 10.3390/app142411750

[ref27] Manikandan S Caroline AL Kanniamma D : The study on clustering analysis in data mining. *International Journal of Data Mining Techniques and Applications.* 2018;7(1):46–49.

[ref28] National Institute of Standards and Technology (NIST) : *Artificial Intelligence Risk Management Framework (AI RMF 1.0).* 2023. 10.6028/NIST.AI.100-1

[ref29] Pala SK : Use and applications of data analytics in human resource management and talent acquisition. *International Journal of Enhanced Research in Science, Technology & Engineering.* 2021;10:2319–7463.

[ref30] Purba W Tamba S Saragih J : The effect of mining data K-Means clustering toward students profile model drop out potential. *IOP Conference Series: Journal of Physics.* 2018;1007(1):012046–012049. 10.1088/1742-6596/1007/1/012049

[ref31] Rawat A Nadavulakere S Isenhour L : Career enhancement strategies, supportive work relationships and subjective career success: The moderating role of family–work conflict. *Career Dev. Int.* 2024;29(4):421–433. 10.1108/CDI-06-2023-0160

[ref32] Rigotti C Fosch-Villaronga E : Fairness, AI & recruitment. *Comput. Law Secur. Rev.* 2024;53:105966. 10.1016/j.clsr.2024.105966

[ref33] Rousseeuw PJ : Silhouettes: A graphical aid to the interpretation and validation of cluster analysis. *J. Comput. Appl. Math.* 1987;20:53–65. 10.1016/0377-0427(87)90125-7

[ref34] Smith SC Todaro MP : *Economic development.* Boston: Pearson Education; 12th ed. 2015.

[ref35] Supriyanti SS Kusmayanti JD Paluseri ARA : Pemberdayaan masyarakat sekitar di wilayah Ibu Kota Nusantara. *Masyarakat Indonesia.* 2023;49(1):93–102.

[ref36] Van der Heijden BIJM Hofer A Semeijn J : “Don’t you worry ’bout a thing” – The moderating role of age in the relationship between qualitative job insecurity and career sustainability. *Career Dev. Int.* 2024;29(5):527–543. 10.1108/CDI-08-2023-0280

[ref37] Venugopal M Madhavan V Prasad R : Transformative AI in human resource management: enhancing workforce planning with topic modeling. *Cogent Bus. Manag.* 2024;11(1):2432550. 10.1080/23311975.2024.2432550

[ref38] Elm E von Altman DG Egger M : The Strengthening the Reporting of Observational Studies in Epidemiology (STROBE) Statement: Guidelines for Reporting Observational Studies. *PLoS Med.* 2007;4(10):e296. 10.1371/journal.pmed.0040296 17941714 PMC2020495

[ref39] Widiyaningtyas T Prabowo MIW Pratama MAM : Implementation of K-Means clustering to distribution of high school teachers. *Proceeding EECSI, Yogyakarta, 19–21 September.* 2017, September;49–54.

[ref40] Widodo SE : *Manajemen pengembangan sumber daya manusia.* Yogyakarta: Pustaka Pelajar;2018.

[ref41] Wiharto W Suryani E : The comparison of clustering algorithms K-Means and Fuzzy C-Means for segmentation retinal blood vessels. *Acta Informatica Medica.* 2020;28(1):42–46. 10.5455/aim.2020.28.42-47 32210514 PMC7085333

[ref42] Zhang M Zhou S Wu Y : Pressure from social media: Influence of social media usage on career exploration. *Career Dev. Int.* 2024;29(1):93–112. 10.1108/CDI-01-2023-0016

