# Supplementary material for: Application of K-Means Clustering for Job Applicant Analysis in Construction Firms Using R
Source: F1000Res. 2026 Jun 16;14:1388. Originally published 2025 Dec 10. [Version 3] doi: 10.12688/f1000research.172383.3 (PMC13294664; doi:10.12688/f1000research.172383.3)
Supplement: Peer review history

# Reviewer report: Reviewer response for version 3

**DOI:** 10.5256/f1000research.201201.r494297

**Published:** 2026-06-26

**Authors:** Olivia Kembuan

**Affiliations:** 1 Universitas Negeri Manado, Sulawesi Utara, Indonesia

I would like to thank the authors for their thorough, thoughtful, and constructive response to my initial review. The revisions made to the manuscript successfully address all of my major and minor concerns. The theoretical framing, methodological transparency, and overall nuance of the conclusions have been significantly improved.

Is the work clearly and accurately presented and does it cite the current literature?

Partly

If applicable, is the statistical analysis and its interpretation appropriate?

I cannot comment. A qualified statistician is required.

Are all the source data underlying the results available to ensure full reproducibility?

No source data required

Is the study design appropriate and is the work technically sound?

Partly

Are the conclusions drawn adequately supported by the results?

Partly

Are sufficient details of methods and analysis provided to allow replication by others?

Yes

Reviewer Expertise:

Data Science

I confirm that I have read this submission and believe that I have an appropriate level of expertise to confirm that it is of an acceptable scientific standard.

# Reviewer report: Reviewer response for version 3

**DOI:** 10.5256/f1000research.201201.r494296

**Published:** 2026-06-24

**Authors:** Deepak Gupta

**Affiliations:** 1 Penn State University, University Park, PA, USA

The authors have made satisfactory amendments.

Is the work clearly and accurately presented and does it cite the current literature?

Partly

If applicable, is the statistical analysis and its interpretation appropriate?

Partly

Are all the source data underlying the results available to ensure full reproducibility?

Partly

Is the study design appropriate and is the work technically sound?

Partly

Are the conclusions drawn adequately supported by the results?

Partly

Are sufficient details of methods and analysis provided to allow replication by others?

Partly

Reviewer Expertise:

Bioprinting, tissue engineering, artificial intelligence

I confirm that I have read this submission and believe that I have an appropriate level of expertise to confirm that it is of an acceptable scientific standard.

# Reviewer report: Reviewer response for version 2

**DOI:** 10.5256/f1000research.196340.r468949

**Published:** 2026-04-20

**Authors:** Ali Pişirgen

**Affiliations:** 1 Karamanoğlu Mehmetbey University, Karaman, Turkey

**Choosing the number of clusters**

 There is an inherent tension: K=3 is motivated by desired recruitment categories, but K should be justified from the data structure as well, otherwise the analysis becomes forced classification by clustering.

 The manuscript should present cluster-number justification more rigorously (e.g., elbow method plus at least one additional diagnostic such as silhouette/gap or stability), and explain that “three clusters” may be a managerial convenience rather than a natural structure.

**Terminology discipline:**

 Use one consistent naming scheme for variables (e.g., “AutoCAD drafting” vs “AutoCAD drawing”; “planning/supervision report writing”). Inconsistencies make reproducibility and interpretation harder, especially if extended data uses different names. Particularly Table 1 and Table 4. Ensure that variable names match

**Reproducibility**

 Because this is an applied observational dataset analysis (not a clinical trial, systematic review, or animal study), CONSORT/PRISMA/ARRIVE are not directly applicable; however, STROBE-style completeness standards are useful for reporting observational data and analysis decisions. ^1^

Is the work clearly and accurately presented and does it cite the current literature?

Yes

If applicable, is the statistical analysis and its interpretation appropriate?

Yes

Are all the source data underlying the results available to ensure full reproducibility?

No source data required

Is the study design appropriate and is the work technically sound?

Partly

Are the conclusions drawn adequately supported by the results?

Yes

Are sufficient details of methods and analysis provided to allow replication by others?

Yes

Reviewer Expertise:

My research focuses on data analytics and decision support systems within the field of information systems, with particular emphasis on machine learning, clustering techniques, and data-driven modeling of socio-economic systems such as scientometrics, tourism, marketing.

I confirm that I have read this submission and believe that I have an appropriate level of expertise to confirm that it is of an acceptable scientific standard, however I have significant reservations, as outlined above.

## References

[1] : The Strengthening the Reporting of Observational Studies in Epidemiology (STROBE) Statement: Guidelines for Reporting Observational Studies. *PLoS Medicine* .2007;4(10) : 10.1371/journal.pmed.0040296 10.1371/journal.pmed.0040296 PMC202049517941714

# Response

**Published:** 2026-05-07

**Authors:** Wahyu Muhammad Ramdhani

**Affiliations:** Educational Research and Evalu, Universitas Negeri Yogyakarta, Yogyakarta, Special Region of Yogyakarta, Indonesia

Dear Reviewer,

 Thank you for your careful and constructive review of our manuscript and for approving the work with reservations. We have revised the manuscript to address each of your comments.

 1. Choosing the number of clusters

 Comment:

 There is an inherent tension: K = 3 is motivated by desired recruitment categories, but K should be justified from the data structure as well. The manuscript should present cluster-number justification more rigorously and explain that three clusters may be a managerial convenience rather than a natural structure.

 Response:

 We agree with this important point. In the revised manuscript, we now explicitly state that the three-cluster solution was selected partly because it corresponded to the organization’s need for an interpretable decision-support structure, but that it should not be interpreted as proof of three naturally distinct applicant groups. We have reframed the three-cluster solution as a managerially interpretable exploratory grouping rather than a definitive natural classification.

 We also expanded the cluster-number justification by discussing internal validity diagnostics, including the elbow method, silhouette coefficient, and Davies–Bouldin Index. The revised manuscript reports a mean silhouette coefficient of 0.16 and a Davies–Bouldin Index of 2.05, which are interpreted cautiously as indicating modest separation rather than strong natural cluster structure.

 Changes made:

 The Methods and Results sections now include clearer discussion of cluster-number justification, managerial interpretability, and internal validity diagnostics. The Results, Discussion, and Conclusions have also been revised to avoid overstating the strength or naturalness of the three-cluster solution.

 2. Terminology discipline

 Comment:

 Use one consistent naming scheme for variables, especially in Table 1 and Table 4. Inconsistencies make reproducibility and interpretation harder.

 Response:

 We agree. We have standardized the terminology across the manuscript, tables, figures, and supplementary materials. The revised manuscript consistently uses the following terms: “AutoCAD drafting skills,” “planning/supervision report-writing skills,” and “adaptability.” We also revised the tables and figure labels to align with this terminology. The previous decision-style labels were replaced with neutral interpretive profile labels to avoid implying automated hiring decisions.

 Changes made:

 Table 1 and the results tables have been revised using consistent terminology. The labels “Rejected,” “Under Consideration,” and “Accepted” were replaced with “Lower competency profile,” “Intermediate/mixed competency profile,” and “Higher competency profile.” Revised figures and supplementary materials were also provided.

 3. Reproducibility and reporting completeness

 Comment:

 Because this is an applied observational dataset analysis, CONSORT/PRISMA/ARRIVE are not directly applicable; however, STROBE-style completeness standards are useful for reporting observational data and analysis decisions.

 Response:

 We agree. We have revised the manuscript to follow STROBE-style completeness principles where relevant. The revised Methods section now clarifies the source of the data, the applicant screening process, the reason for analysing only the 30 shortlisted candidates, the assessment variables, preprocessing decisions, clustering parameters, and limitations of inference.

 Changes made:

 We added explicit explanation of the analytical sample, eligibility process, preprocessing decisions, and reporting boundaries. We also cited the STROBE statement and revised the limitations section to clarify the observational and exploratory nature of the study.

 4. Use of supplementary materials

 Response:

 We also updated the supplementary materials to improve consistency and reproducibility. The revised extended data include the final R script, clustering outputs, revised figure files, and supporting documentation. The supplementary terminology was updated to match the revised manuscript.

 We appreciate the reviewer’s comments, which helped us improve the methodological discipline, terminology consistency, and interpretation of the clustering results.

# Reviewer report: Reviewer response for version 2

**DOI:** 10.5256/f1000research.196340.r467509

**Published:** 2026-03-23

**Authors:** Olivia Kembuan, Ferdinan Sangkop

**Affiliations:** 1 Universitas Negeri Manado, Sulawesi Utara, Indonesia; 2 informatics, Universitas Negeri Manado (Ringgold ID: 175496), Tondano, North Sulawes, Indonesia

**Is the work clearly and accurately presented and does it cite the current literature?**

 The manuscript is generally clearly structured and readable.  However, the literature review focuses primarily on clustering algorithms and includes a number of regional or context-specific references. The manuscript would benefit from stronger engagement with more recent international literature on HR analytics, algorithmic decision-making in recruitment, and AI-assisted hiring systems. Incorporating broader global scholarship would strengthen the theoretical grounding of the study.

**Is the study design appropriate and is the work technically sound?**

 Provide a clearer justification for analyzing only the 30 shortlisted candidates and discuss the implications of this sampling decision.

 Although some methodological details are provided, additional clarification of analytical parameters and preprocessing decisions would improve reproducibility.

**Are the conclusions drawn adequately supported by the results?**

 The conclusions generally align with the exploratory nature of the study; however, some claims regarding the usefulness of clustering for improving recruitment decision-making are somewhat stronger than the evidence supports. Because the analysis is based on a small, pre-screened sample and lacks external validation against actual hiring outcomes or job performance, the conclusions should emphasize the illustrative and exploratory nature of the findings rather than suggesting demonstrated improvements in recruitment effectiveness.

Is the work clearly and accurately presented and does it cite the current literature?

Partly

If applicable, is the statistical analysis and its interpretation appropriate?

I cannot comment. A qualified statistician is required.

Are all the source data underlying the results available to ensure full reproducibility?

No source data required

Is the study design appropriate and is the work technically sound?

Partly

Are the conclusions drawn adequately supported by the results?

Partly

Are sufficient details of methods and analysis provided to allow replication by others?

Yes

Reviewer Expertise:

Data Science

We confirm that we have read this submission and believe that we have an appropriate level of expertise to confirm that it is of an acceptable scientific standard, however we have significant reservations, as outlined above.

# Response

**Published:** 2026-05-07

**Authors:** Wahyu Muhammad Ramdhani

**Affiliations:** Educational Research and Evalu, Universitas Negeri Yogyakarta, Yogyakarta, Special Region of Yogyakarta, Indonesia

Dear Reviewers,

 Thank you for your constructive evaluation of our manuscript and for approving the work with reservations. We have revised the manuscript substantially to address your comments.

 1. Engagement with broader international literature

 Comment:

 The literature review focused primarily on clustering algorithms and included several regional or context-specific references. The manuscript would benefit from stronger engagement with more recent international literature on HR analytics, algorithmic decision-making in recruitment, and AI-assisted hiring systems.

 Response:

 We agree with this comment. In the revised manuscript, we have expanded the literature review by adding a new discussion of HR analytics, algorithm-assisted recruitment, AI-assisted hiring, fairness, transparency, explainability, adverse impact, and human oversight. We incorporated broader international literature and governance-oriented sources to strengthen the theoretical grounding of the study. The revised manuscript now positions K-Means clustering within the wider context of responsible recruitment analytics and decision support rather than only within clustering methodology.

 Changes made:

 We revised the Introduction and added a new literature review subsection on HR analytics and algorithm-assisted recruitment. We also added recent international references on AI-assisted recruitment, HR analytics, algorithmic fairness, and AI governance.

 2. Justification for analysing only 30 shortlisted candidates

 Comment:

 Provide a clearer justification for analyzing only the 30 shortlisted candidates and discuss the implications of this sampling decision.

 Response:

 We agree. The revised manuscript now explains that 161 applicants initially applied, but only 30 candidates passed the company’s document-screening procedure and completed in-person competency testing. Only these 30 candidates had complete scores for all three assessment variables. We have clarified that this is a pre-screened analytical sample and that the findings should not be generalized to the full applicant pool or to construction applicants more broadly.

 Changes made:

 The Methods section now includes a clearer explanation of the screening procedure, the reason for limiting the analysis to 30 candidates, and the implications of this sampling decision for interpretation and generalizability.

 3. Clarification of analytical parameters and preprocessing decisions

 Comment:

 Additional clarification of analytical parameters and preprocessing decisions would improve reproducibility.

 Response:

 We have revised the Methods section to clarify the preprocessing and clustering workflow. The revised manuscript states that all variables were measured on the same 0–100 scale, that raw scores were retained, that no additional normalization was applied, and that Euclidean squared distance was used in the clustering procedure. We also clarified that detailed clustering outputs and supporting materials are provided as extended data.

 Changes made:

 We revised the Methods section on assessment variables, preprocessing, outlier/sensitivity checks, and clustering procedure. We also updated the supplementary materials to align with the revised manuscript terminology.

 4. Claims regarding usefulness of clustering for recruitment decision-making

 Comment:

 Some claims regarding the usefulness of clustering for improving recruitment decision-making were stronger than the evidence supports. Because the analysis is based on a small, pre-screened sample and lacks external validation against actual hiring outcomes or job performance, the conclusions should emphasize the illustrative and exploratory nature of the findings.

 Response:

 We agree and have revised the manuscript accordingly. The revised version no longer presents clustering as evidence of demonstrated improvement in recruitment effectiveness. Instead, clustering is framed as an exploratory and illustrative decision-support technique for organizing applicant assessment data. We explicitly acknowledge that the analysis was based on a small, pre-screened sample and was not externally validated against hiring outcomes or job performance.

 Changes made:

 We revised the Abstract, Results, Discussion, Limitations, and Conclusions to emphasize the exploratory nature of the findings. We also added explicit limitations regarding sample size, pre-screening, lack of external validation, and the need for future studies using larger datasets and job-performance outcomes.

 We appreciate the reviewers’ comments, which helped us strengthen the theoretical framing, methodological transparency, and cautious interpretation of the manuscript.

# Reviewer report: Reviewer response for version 1

**DOI:** 10.5256/f1000research.190104.r446627

**Published:** 2026-02-03

**Authors:** Deepak Gupta

**Affiliations:** 1 Penn State University, University Park, PA, USA

The manuscript addresses a relevant and practically important topic of using K‑Means clustering to support recruitment decisions in a construction consulting firm. The use of real organizational data, clear cluster descriptions, and intuitive 2D/3D visualizations makes the work accessible and potentially useful for practitioners interested in HR analytics and R-based implementations. However, the current version has substantial methodological and presentation limitations that need to be addressed before the article can be considered for indexing.

1. The study starts from 161 applicants but analyzes only 30 shortlisted candidates who “met the minimum requirements,” with no clear justification of the selection criteria or their implications. This design means the clustering is applied to a highly pre-filtered subset rather than the full applicant pool, which severely limits generalizability to “recruitment strategies” or “workforce selection” more broadly. The authors should explicitly describe the screening rules used to select the 30 candidates (cut‑offs, qualitative judgments, etc.). The authors should re-run the analysis on the full set of 161 applicants (if possible and ethically permissible) or reframe the paper as a methodological demonstration on a small, filtered sample and tone down claims about recruitment optimization.
2. Initial centroids are chosen manually as “Rejected = (60,75,85); Considered = (62,77,88); Accepted = (70,84,92)” with no methodological justification beyond being “chosen randomly,” which they are not. These values embed prior expectations about the three groups and risk steering the solution toward a desired structure, rather than letting the algorithm discover patterns in the data. The authors should justify the chosen centroids and make that explicit. In addition, run at least one standard initialization strategy (e.g., built‑in k-means in R with multiple random starts, or k-means++). The authors should also report whether the manually initialized solution is stable across different initializations; and if not, the conclusions about three clusters and their interpretation need to be more cautious.
3. The methods mention Euclidean distance and cite general clustering references, but there is no clear statement on whether the three variables were standardized or used in raw form. There is also no discussion of data cleaning beyond a vague reference to removing “irrelevant or incomplete data.” How many records, if any, were excluded and why? Whether any outliers were identified and how they were handled. The authors should explicitly state whether they standardized the variables before clustering; if not, justify why not and show at least descriptive ranges/variances. They should also provide a proper description of data-cleaning procedure. If outliers exist (some points in the 3D plot appear distant from cluster centers), please clarify whether they influence centroid placement and consider sensitivity analyses.
4. The discussion and conclusions repeatedly assert that K‑Means is “effective” and “improves recruitment decision-making,” yet there is no external validation. There is no comparison with actual hiring decisions made by the firm. No HR-expert assessment of whether clusters align with real performance or selection outcomes. If such data are not available, please qualify all claims about decision-making benefits as exploratory.
5. The author states in the “Underlying data” section that the dataset is not publicly available due to confidentiality and must be requested via the corresponding author. At the same time, the text refers to R scripts and supplementary materials in a Zenodo repository as extended data. The authors should clarify precisely what is in the Zenodo archive (R code, example data, figures, documentation) and referencing it consistently in the methods and data availability sections. If contractual and legal constraints allow, please consider publishing an anonymized dataset (at least the 30 shortlisted candidates; ideally all 161) with no identifying information. If this is impossible, explicitly justify the restriction and explain how others can still verify the methodology (e.g., via synthetic data included in the Zenodo package).
6. The conclusions and parts of the discussion are phrased quite strongly (e.g., “confirm that the K‑Means Clustering algorithm is an effective tool” and “this approach not only addresses challenges in evaluating applicants but also builds a foundation for sustainable talent management”), which is not fully justified given the small, preselected sample and lack of external validation.
7. Minor comments: The authors are suggested to modify the text in the abstract, discussion, and conclusions to reflect that findings are preliminary and illustrative for one firm and one small dataset.
8. The literature review is generally coherent but heavily focused on clustering and Indonesian/sector-specific references. There is relatively little engagement with the broader international work on AI in recruitment, algorithmic decision-making, or HR analytics.
9. Tables 2-7 list centroid updates and distances in excessive detail. It is pedagogically useful but makes the manuscript long and difficult to navigate. Some of the tables can be moved to the supplementary information with their summary in the main text.
10. There are some grammatical errors, please perform a proofread of the manuscript carefully.

Is the work clearly and accurately presented and does it cite the current literature?

Partly

If applicable, is the statistical analysis and its interpretation appropriate?

Partly

Are all the source data underlying the results available to ensure full reproducibility?

Partly

Is the study design appropriate and is the work technically sound?

Partly

Are the conclusions drawn adequately supported by the results?

Partly

Are sufficient details of methods and analysis provided to allow replication by others?

Partly

Reviewer Expertise:

Healthcare, 3D Printing, Artificial Intelligence

I confirm that I have read this submission and believe that I have an appropriate level of expertise to confirm that it is of an acceptable scientific standard, however I have significant reservations, as outlined above.

# Response

**Published:** 2026-02-06

**Authors:** Wahyu Muhammad Ramdhani

**Affiliations:** Educational Research and Evalu, Universitas Negeri Yogyakarta, Yogyakarta, Special Region of Yogyakarta, Indonesia

Response to Reviewer 2

**Deepak Gupta (Penn State University, USA)**

 We thank the reviewer for the detailed and technically informed evaluation of our manuscript. The feedback has been instrumental in improving methodological rigor, transparency, and interpretive clarity. Our responses are provided below.

**Comment 1: Pre-filtered sample and limited generalizability**

**Response:**

 We fully agree. The manuscript has been explicitly reframed to clarify that the clustering analysis was applied to a *pre-screened subset* of applicants who passed document screening. This design choice is now clearly justified in the Methods section, and all claims have been moderated accordingly. The study is presented as an *exploratory analytical illustration* rather than a full recruitment optimization model.

**Comment 2: Manual centroid bias and need for robustness checks**

**Response:**

 This concern has been directly addressed. In addition to clarifying the rationale for initial centroid selection, we now report results from **multiple random initializations** (nstart = 50) using R’s kmeans() function. The three-cluster structure remained largely stable across runs, with only minor variation among borderline profiles. These findings are now reported and discussed in the revised manuscript.

**Comment 3: Data normalization, outliers, and cleaning**

**Response:**

 The Methods section now explicitly states that all variables were measured on the same 0–100 scale, and therefore raw scores were used without normalization. We also added an **outlier and sensitivity analysis**, including a leave-one-out test removing the most distant observation. This test did not materially change the cluster structure, and validation metrics showed only marginal changes. These results are now reported to support robustness.

**Comment 4: Lack of validation against real hiring outcomes**

**Response:**

 We explicitly acknowledge this limitation. The revised manuscript clearly states that no comparison with actual hiring decisions or post-employment performance was possible. All statements regarding recruitment support are now framed as *illustrative and exploratory*, not empirically validated improvements.

**Comment 5: Data availability and Zenodo clarification**

**Response:**

 This issue has been resolved by clearly distinguishing:

- Confidential underlying data (not publicly shareable);
- Publicly available extended data hosted on Zenodo, including R scripts, figures, clustering outputs, and documentation.

The Data availability section has been rewritten for clarity and compliance with journal policy.

**Comment 6: Excessive tables and manuscript length**

**Response:**

 In line with the suggestion, detailed centroid iteration tables have been moved to **extended data**. The main text now focuses on final clustering outcomes, visual interpretation, and discussion, substantially improving readability.

**Comment 7: Language and proofreading**

**Response:**

 The manuscript has been carefully proofread and revised to improve clarity, reduce repetition, and ensure consistent terminology throughout.

**Final note:**

 We thank the reviewer for the rigorous and constructive critique. The revisions have significantly strengthened the manuscript’s technical soundness, transparency, and responsible positioning within data-driven recruitment research.

# Reviewer report: Reviewer response for version 1

**DOI:** 10.5256/f1000research.190104.r446632

**Published:** 2026-01-08

**Authors:** Sonia Najam Shaikh

**Affiliations:** 1 Jiangsu University, Zhenjiang, China

- The paper presents an interesting and practically valuable idea by using K-Means clustering to support recruitment decision-making in a construction firm. The study groups applicants into rejected, under consideration, and accepted categories based on AutoCAD skills, planning/supervision report ability, and adaptability, and it makes good use of R and visualization techniques to demonstrate the results. The topic is timely and relevant to HR analytics and industry needs, and overall the manuscript is readable, logically structured, and supported by real organizational data, which gives it strong applied significance.
- However, while the work is clearly presented, it is only partly sufficient in terms of scientific rigor and presentation quality. The manuscript sometimes becomes repetitive and uses very long tables that make it difficult to follow the narrative smoothly. The literature review is decent, but it leans heavily on regional or context-specific studies, and it would benefit greatly from integrating more current international literature on AI-driven recruitment, algorithmic hiring, fairness, and HR analytics trends. Adding such perspectives would make the study more globally relevant and intellectually grounded.
- In terms of study design, the concept is appropriate, but there are some important weaknesses that affect technical soundness. The biggest issue is that the study only analyzed 30 shortlisted candidates out of 161 applicants, without providing a clear justification. This introduces bias and limits how generalizable the findings are. Another concern is the selection of initial centroids, which appears subjective rather than determined using a standard procedure such as random initialization or k-means++. There is also no explanation of whether data normalization was applied, which is important since K-Means relies on Euclidean distance and differences in scale can significantly affect results. These issues really need to be addressed for the paper to be scientifically strong.
- The paper partly allows replication because the clustering process is explained carefully and tables clearly trace centroid iterations and cluster formation. However, critical methodological details are missing. There is no detailed explanation of data cleaning, whether any applicants were excluded and why, how outliers were treated, whether ethical permission or company approval was obtained, and what settings were used in R. To make the study convincingly reproducible, the authors should provide anonymized raw data, the R script, and a clearer explanation of all methodological choices.
- The analysis and interpretation are also only partly sufficient. Although clustering has been correctly applied and visualizations are very good, the study does not present objective validation of the clusters. There is no numerical reporting of validation metrics such as silhouette score, Davies–Bouldin index, or any form of comparison with real hiring outcomes or expert HR evaluation. Without such validation, the findings are mostly descriptive, and the claim that clustering improves recruitment decision-making is not empirically proven. Adding cluster validity results and some form of comparison or evaluation would significantly strengthen the credibility of the conclusions.
- Data availability is partly fulfilled. While applicant scores are shown in tables, there is no formal dataset sharing, no supplementary files, and no data availability statement. Reproducibility is an important expectation, so providing anonymized data and the analysis code will help readers trust and build upon the work.
- The conclusions are relevant and aligned with the results, but they are currently expressed too strongly considering the methodological limitations and lack of validation. The study needs to acknowledge its limitations, including the small selective sample, potential bias, lack of external validation, and ethical concerns such as fairness and transparency in automated hiring. The tone of the conclusions should be slightly moderated to reflect that the findings are promising but not definitively proven.
- Overall, this is a promising and meaningful study with clear practical relevance, strong visualization, and a good foundation. However, to make it scientifically sound and suitable for indexing, the authors must justify or expand the sample, apply standard centroid initialization or explain theirs clearly, normalize or justify scale handling, provide quantitative cluster validation, share data and code, strengthen literature support, and include a more explicit discussion of limitations and ethical considerations. 

Is the work clearly and accurately presented and does it cite the current literature?

Partly

If applicable, is the statistical analysis and its interpretation appropriate?

Partly

Are all the source data underlying the results available to ensure full reproducibility?

Partly

Is the study design appropriate and is the work technically sound?

Partly

Are the conclusions drawn adequately supported by the results?

Partly

Are sufficient details of methods and analysis provided to allow replication by others?

Partly

Reviewer Expertise:

Human Resource Management, Artificial intelligence, Big data analytics capability

I confirm that I have read this submission and believe that I have an appropriate level of expertise to confirm that it is of an acceptable scientific standard, however I have significant reservations, as outlined above.

# Response

**Published:** 2026-02-06

**Authors:** Wahyu Muhammad Ramdhani

**Affiliations:** Educational Research and Evalu, Universitas Negeri Yogyakarta, Yogyakarta, Special Region of Yogyakarta, Indonesia

Response to Reviewer 1

**Sonia Najam Shaikh (Jiangsu University, China)**

 We sincerely thank the reviewer for the careful reading of our manuscript and for the constructive and detailed feedback. We have revised the manuscript substantially to address all major concerns raised. Our point-by-point responses are provided below.

**Comment 1: Limited international literature and lack of broader AI/HR analytics perspective**

**Response:**

 Thank you for this important suggestion. We have expanded the literature review and discussion to better situate the study within international debates on data-driven recruitment, employability, and HR analytics. Specifically, we incorporated recent global literature addressing analytical decision support, adaptability, and career sustainability (e.g., Akkermans et al., 2024; Donald et al., 2024; Rawat et al., 2024; Zhang et al., 2024; Van der Heijden et al., 2024).

 In addition, we explicitly positioned clustering as an *exploratory decision-support tool*, aligned with international guidance on responsible and transparent use of analytics in employment contexts.

**Comment 2: Analysis limited to 30 candidates out of 161 applicants**

**Response:**

 We fully agree that this required clearer justification. The revised manuscript now explicitly explains that the clustering analysis was conducted on 30 candidates who passed formal document screening conducted by the company as part of standard recruitment procedures. We clearly state that the study analyzes a *pre-screened assessment sample*, not the full applicant pool.

 Accordingly, the study has been reframed as an *exploratory methodological illustration* rather than a comprehensive recruitment optimization model. Limitations related to selection bias and generalizability are now explicitly acknowledged in the Methods, Results, and Conclusions sections.

**Comment 3: Subjective centroid selection and lack of standard initialization**

**Response:**

 Thank you for highlighting this critical methodological issue. We have clarified that the manually specified initial centroids were derived from preliminary inspection of score distributions and used for transparency and pedagogical traceability.

 To address concerns regarding subjectivity and robustness, we have now:

1. Added a dedicated subsection describing **multiple random initializations** using the built-in kmeans() function in R (nstart = 50);
2. Reported **stability checks**, showing that the three-cluster structure was largely consistent across runs, with only minor variations among borderline cases;
3. Moderated all interpretations to emphasize that clusters reflect *similarity patterns*, not predefined hiring categories.

**Comment 4: Missing details on data cleaning, ethics, and reproducibility**

**Response:**

 This concern has been fully addressed. The revised manuscript now includes:

- A clearer description of document screening and data preprocessing;
- Explicit confirmation that all analyzed records were complete and measured on the same 0–100 scale;
- A detailed **Ethical approval and informed consent** section clarifying the use of fully anonymized secondary data under a formal Data Usage Agreement;
- An updated **Data availability statement**;
- An updated **Zenodo repository** containing R scripts, clustering outputs, and documentation to support reproducibility.

**Comment 5: Lack of objective cluster validation**

**Response:**

 We agree with this observation. To strengthen technical rigor, we have added a dedicated **cluster validity assessment** section reporting internal validation metrics. Specifically, we report:

- Mean silhouette coefficient (0.16);
- Davies–Bouldin Index (DBI = 2.05).

We explicitly interpret these values as *diagnostic indicators* of modest separation, appropriate for exploratory analysis, and avoid presenting them as evidence of predictive effectiveness.

**Comment 6: Overly strong conclusions**

**Response:**

 The Conclusions section has been revised to adopt a more cautious and balanced tone. Claims regarding effectiveness and decision-making improvement are now framed as *exploratory and context-specific*. Limitations related to sample size, pre-screening, lack of external validation, and ethical considerations are explicitly acknowledged.

**Final note:**

 We sincerely appreciate the reviewer’s thoughtful comments, which have significantly strengthened the manuscript’s methodological transparency, analytical caution, and international relevance.
